# Modulating Electrostatic Interactions to Control the Analyte Transport in Nanochannels

**DOI:** 10.1021/acsami.5c14563

**Published:** 2025-10-06

**Authors:** H. Samet Varol, Matteo Cingolani, Francesco Casnati, Damiano Genovese

**Affiliations:** Dipartimento di Chimica “Giacomo Ciamician”, Università di Bologna, via Selmi 2, Bologna 40126, Italy

**Keywords:** ionic diffusion, nanoconfinement, fluorescence correlation spectroscopy, membrane

## Abstract

Ion-receptor binding is a key mechanism underlying various biological responses, which greatly inspires biomimetic approaches in technologies ranging from nanomedicine to energy storage and active membrane separation. Interaction between analytes and nanopores has been reported to either favor the transport (electrochemical studies performed in the millimolar concentration regime) or to slow down the diffusion in nanochannels (single-molecule investigations in the nanomolar range). Here, we propose a simple and inexpensive fluorescence setup for monitoring submicromolar diffusion, which effectively bridges these two concentration regimes, and show that at micromolar concentration, electrostatic interactions between the analyte (Ru­(bpy)_3_^2+^) and nanochannel walls slow down the transport by ca. 20% due to the diffusion mediated by transient surface adsorption. The occurrence of this mechanism has been previously investigated using single-molecule FCS techniques, and it is confirmed here, even in bulk measurements conducted at micromolar concentrations. Furthermore, we demonstrate that electrostatic interactions can be (i) switched off by changing the pH to acidic, or can be (ii) finely tuned by adding a competitor divalent cation (Ca^2+^), which effectively competes with the cationic analyte (Ru­(bpy)_3_^2+^) for the negatively charged walls, allowing smoother diffusion through the nanochannels.

## Graphical abstract

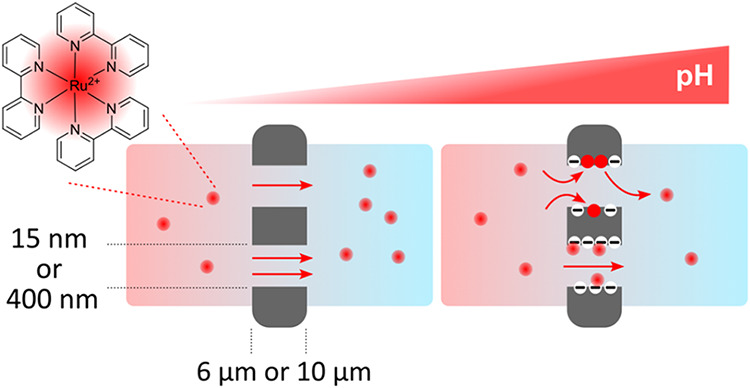

## Introduction

Solid-state nanoporous membranes have garnered considerable interest due to their high surface area-to-volume ratio, finely tunable pore geometry and chemistry, as well as being fabricated from a wide range of materials, including polymers, inorganic materials (e.g., silicon, silica, and alumina), and hydrogels.^1,2^ These membranes’ structural and chemical advantages make them feasible for different applications in catalysis, engineering, drug delivery, sensing and detection, and energy conversion and storage.^3,4^ For such applications to be effective, ion transport in a nanoconfined space and nanopore activity should be precisely controlled. In this context, ion-track-etched multi- and nanoporous polymeric membranes hold a unique position due to their mechanical and chemical durability, finely tunable nanopore (more precisely, nanochannel) size, density, geometry, and length.^5–7^ Beyond their structural advantages, the surface groups of such track-etched nanochannels also make them responsive against various stimuli, such as environmental pH,^8–10^ and allow them to be chemically modified by various molecules such as polyelectrolytes,^11,12^ inorganic crystals,^13–16^ or stimuli-responsive polymer brushes.^17,18^ Combining all their structural properties with chemical surface modifications makes the ion-track-etched membranes a unique tool for sorting ionic nanochannel transport for complex engineering applications.

During the past few years, solid-state nanoporous membranes have received significant interest in energy storage and conversion applications thanks to their control over *cation transport and selectivity*,^6,19–21^ and precise control of nanopore cation activities has been applied to ion sieving applications,^6^ with higher than 1000 selectivity factor recently obtained for various monovalent/divalent cation sieving.^13^ One key issue in controlling the ionic nanopore transport is the diameter of the nanochannels where the ions travel or are selected. Nanoconfinement arises in nanochannels with a sufficiently narrow diameter (<20 nm), where the transport behavior starts to diverge from a bulk conductance to permselectivity due to the contribution of the electron double layer (EDL) structure inside the pores.^22^ When the pore opening gets very small (a couple of nanometers) and the EDL layer gets very close (Debye screening length of ions), electrostatic attraction/rejection between EDL and analyte defines the nanopore transport of the analyte. However, it is still an interesting question if EDL can still affect the ion nanopore transport even if the pore opening is far beyond the Debye screening length of the ions, where the ion transport is in the bulk conductance range (diameters of tens to hundreds of nm).

To date, the advanced ionic transport of single and multipore ion-track-etched membranes has generally been characterized via electrochemical probing techniques such as ionic current measurements and cyclic voltammetry, which provide real-time monitoring of ion transport under applied voltages.^7,23^ Another popular probing method is the osmotic pressure-driven transport setups—diffusion setups—combined with different photophysical characterization techniques such as UV–vis or fluorescence spectroscopy and microscopy.^2,8,9,24–26^ These setups allow the direct observation of concentration-driven ionic transport without external stimuli, making them ideal for investigating fundamental transport mechanisms in various nanochannel systems. To date, electrochemical or photophysical probing techniques for studying ionic transport inside polymeric nanochannels have primarily focused on diffusion studies at high ionic analyte concentrations (in the millimolar range).^8,9,25^ At these concentrations, electrostatic interactions between charged nanopore surfaces and analytes are often screened by high ionic strength, enhancing the permeability. For instance, studies have shown that negatively charged nanopores (e.g., at high pH) can electrostatically attract cationic analytes, enhancing transport rates through charge-selective transport and permselectivity in synthetic nanochannels.^9,25^ Besides electrostatic rejection and attraction between EDL and analyte, also (i) size exclusion and (ii) hydrophobic interactions between analyte and nanopore walls are the other two major factors playing a direct role in ionic transport.^8,27^ Electrostatic attraction/repulsion alone may not fully explain ion (analyte) transport at low analyte concentrations, where analyte–pore wall interactions become dominant and less influenced by bulk ionic strength.^2,28–30^ At a few μM or lower concentrations, factors such as electrostatic and chemical interactions, reversibility or irreversibility of adsorption, pH effects on the ionic strength in the EDL, and pore wetting significantly impact transport. Moreover, nanopore/nanochannel studies at low analyte concentrations are crucial for applications like single-molecule DNA sequencing,^30,31^ biomarker detection,^32^ and environmental sensing of pollutants and heavy metals.^33^ Single-molecule sensitive techniques, such as fluorescence correlation spectroscopy (FCS), operate in the nanomolar to micromolar range, enabling precise monitoring of nanopore activities such as ion trapping, transient binding events, and hopping mechanisms, but remain limited by low analyte availability.^29,30,34^

Bioinspired ligand-binding techniques regulate ionic transport in synthetic nanopores by binding competitive ions (e.g., Ca^2+^, Mg^2+^) to charged pore walls, controlling analyte permeability.^35–37^ This approach has been well studied, but systematic investigations into the direct interaction between ionic analytes and nanopore walls remain limited. Such interactions—driven by electrostatic forces, hydrogen bonding, and hydrophobic effects—play a crucial role in confined transport, especially at low analyte concentrations.^29^ Recent studies suggest transient analyte adsorption along pore walls influences diffusion,^38–42^ yet its impact on concentration-driven transport across different nanoconfinements remains an open question.

In this work, we investigate how the cationic luminescent analyte Ru­(bpy)_3_^2+^ interacts with negatively charged nanochannels in multiporous polycarbonate ion-track-etched membranes at low concentrations and how these interactions influence its transport under different pH conditions. Specifically, we examine the combined effects of nanoconfinement, environmental pH, and analyte-nanopore interactions on the diffusion dynamics. We further explore the reversibility of these interactions and the role of competitive ion binding (Ca^2+^) in modulating transport. Our findings provide new insights into ionic analyte-nanopore interactions in nanofluidic systems with broader implications for selective ion transport, sieving mechanisms, and ionic sensing technologies.

## Experimental Section

### Materials

Tris­(2,2′-bipyridyl)­ruthenium­(II) chloride (Ru­(bpy)_3_^2+^), calcium chloride (CaCl_2_), sodium chloride (NaCl), and 99.8% pure ethanol (EtOH) were purchased from Sigma-Aldrich and used without any further purification. Whatman Nuclepore Track-Etched polycarbonate (PC) membranes with different pore diameters were purchased from Cytiva (Sigma-Aldrich): 400 nm (called the *Large Nanochannel (LNC) Membrane*, WHA10417118), and 15 nm (called the *Small Nanochannel (SNC) Membrane*, WHA110601). Further characteristics of the track-etched membranes are listed in Table 1. The PBS solutions were prepared by using tablets (one tablet in 200 mL solution results in 1× PBS solution) purchased from Merck. Water (MiliQ)-based hydrogen chloride (HCl, 1 M) and Sodium hydroxide (NaOH, 1 M) solutions were added dropwise to adjust the pH of the analyte solutions.

**Table 1:** Summary of the Critical Structural Dimensions of Our Membranes for Ionic Nanopore Diffusion

		***SNC membrane*** ^a^	***LNC membrane***
pore size (nm)	permeate side	15 nm	372 ± 29 nm
	feed side	15 nm	376 ± 18 nm
pore density (pores/cm^2^)		6 × 10^8^	10^8^
membrane film thickness		6.5 ± 0.6 μm	9.4 ± 0.5 μm
total pore opening for ionic diffusion (cm^2^)		5.32 × 10^–4^	6.31 × 10^–2^

a Presented values of SNC membranes, except the membrane film thickness, were obtained from the producer.

### Scanning Electron Microscopy (SEM) Imaging

For the cross-sectional SEM imaging of the membranes, they were torn while being submerged in liquid nitrogen and then fixed to an aluminum sample holder using double-sided carbon tape. Before SEM imaging, the surface of the membranes was sputter-coated with ca. 2 nm thick Au during 1 min and at 30 mA current conditions (Edwards S150B).^43^ The Au-coated membranes were then imaged using a Leica/Cambridge Stereoscan 360 type (Oxford Instruments) SEM microscope at an acceleration voltage (EHT) of 6 kV, and at a working distance of 8 mm. The current during the imaging was fixed at 23 pA.

### Static Contact Angle (CA_static_) Measurements

3 μL sessile water drops were left on membrane surfaces, and then droplets in contact with the membrane were recorded for 10 s by a KSV CAM101 instrument (KSV Instruments Ltd., Helsinki, Finland). Imaged droplets in contact with surfaces were subsequently analyzed using a Young–Laplace fitting using the instrument’s software.^44^ Before the sessile drop addition, the membranes were hung in air using glass spacers to prevent any possible capillary forces from acting on the results. A minimum of 3 different measurements were performed for each reported CA_static_.

### Automated Measurement of Cationic Mass Transport (Diffusion) at Membrane Nanochannels

The custom-built ionic diffusion setup (illustrated in Figure 1d) consists of two equal Teflon (Polytetrafluoroethylene, PTFE) cells with magnetic stirrers inside, a peristaltic pump (Watson Marlow 323s/D), silicone and fluorinated ethylene propylene (FEP) tubes, and a 3-in-1 quartz flow-through cell (550 μL inner volume, 10 × 5 mm optical path length, Hellma) connected to a Horiba FluoroMax-4 type spectrofluorometer (Horiba, Edison, NJ). The PC membrane was initially positioned between the two Teflon cells, fully covering the diffusion channel area of the cells (8 mm diameter, 0.502 cm^2^) and the opaque slide facing toward the cell, where the analyte was later placed (called the *feed cell*). The second Teflon cell, where the analyte diffuses through the membrane and gathers, is called the *permeate cell*. Subsequently, 4918.5 and 3243.5 μL of 1× PBS solution (without analyte) with a fixed pH (pH 2.5 or pH 7) were added to the permeate and feed cells, respectively. The 1668.5 μL volume difference is due to the solution in the tubes and the flow-through cuvette connected to the setup’s permeate cell. During the entire diffusion experiment, the peristaltic pump circulated the solution between the permeate cell and the flow-through cuvette (placed inside the spectrofluorometer) at a speed of 120 rpm. The spectrofluorometer operated in kinetic mode, with 35 cycles (unless otherwise mentioned in the text) of 6 s and a time delay of 354 s. The set excitation (exc) and emission (em) parameters were: λ_exc_ = 450 nm, slit opening_ext_ = 2 nm, λ_em_ = 608 nm, and slit opening_em_ = 2 nm. Fluorescence signal intensity at 608 nm was saved throughout the (3.5 h) diffusion experiment. To ensure nanochannel wetting, *conditioning*, the first 5 cycles of measurements were collected before any analyte addition to the feed solution. The dye was introduced into the feed cell (5 μM initial analyte concentration) immediately after collecting the fifth measurement. To fix the initial feed concentration of the analyte to 5 μM, 6.5 μL from the analyte stock solution (2.5 mM Ru­(bpy)_3_^2+^) was added to the feed cell while adding the same amount of 1× PBS solution (no dye) in the permeate cell. This protocol has been followed for each experimental condition for a specific membrane as a minimum of three replicates to obtain statistically significant ionic diffusion results. The data collected from the spectrofluorometer were analyzed by using data processing software Origin Pro 9.0 and Igor Pro.

**Figure 1:**
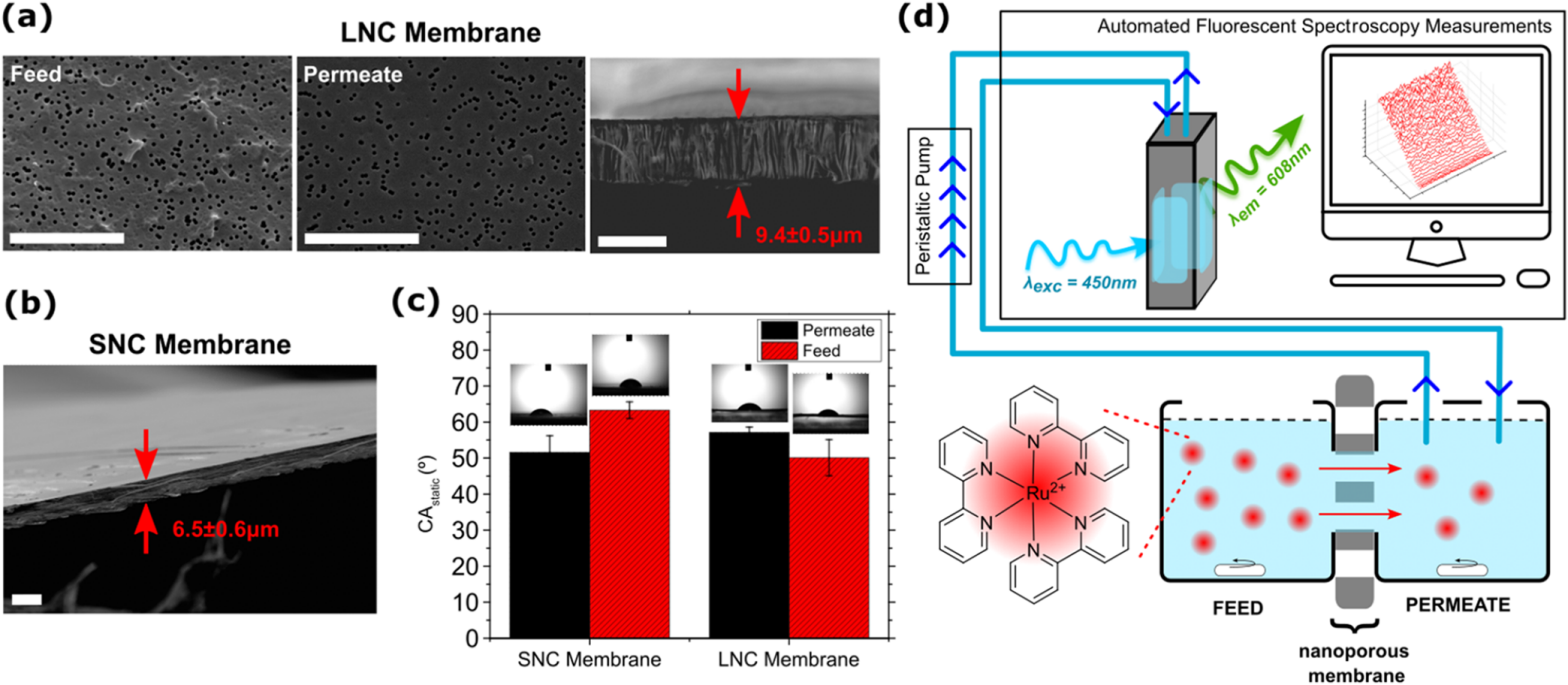
SEM micrographs of (a) feed (left) and permeate (middle) faces and (right) cross section of the large nanochannel membrane (*LNC Membrane*). (b) Cross-sectional image of the small nanochannel membrane (*SNC Membrane*). (a-right and b) Membranes’ thickness (between red arrows) is noted inside the images (avg ± SD). Scale bars are 10 μm. (c) CA_static_ results were collected from the feed and permeate sides of the small and large nanochannel membranes. Photographic images were taken while performing the CA_static_ experiments; 3uL sessile drops were in contact with the membranes. Error bars are SD. (d) Illustration of the automated ionic diffusion setup used for this work.

### Cationic Mass Transport in the Presence of a Competitor Divalent Cation (CaCl_2_)

Despite the differences indicated here, the rest of the experimental protocol was kept identical as indicated in the automated measurement of cationic mass transport (diffusion) at membrane nanochannels submethods section. For these measurements, inside the feed solution (prior to any Ru­(bpy)_3_^2+^ addition), different amounts of CaCl_2_ (2.5, 5, 7, 20 or 50 mM) were added. After the pH of the solution was adjusted, it was placed in the feed chamber. At the same time, the permeate chamber was filled with pristine 1× PBS solution at the same pH as the solution in the feed container. Similar to the methods in the previous section, after 30 min of conditioning, Ru­(bpy)_3_^2+^ dye was added to the feed solution. The fluorescence signal was recorded as described above during the entire conditioning and diffusion stages (3.5 h).

### Conversion of Fluorescence Intensity to Normalized Analyte–Nanochannel Transport (nmol·cm^–2^)

The calibration line presents the relationship between the analyte (Ru­(bpy)_3_^2+^) amount and the fluorescence signal intensity (unit: CPS) of the emission peak at 608 nm was constructed for pH 2.5 and pH 7 (Figure S3b). A general relationship between the fluorescence intensity and the molar quantity (nmol) was established using a linear fit to the calibration curve:

x=(y−b)/a 1

where *x* is in nmol, *y* is the luminescence intensity in CPS, b is the *y*-intercept, and a is the slope (in nmol^–1^). *x* was then divided by the membrane area where the analyte contacts the membrane (0.502 cm^2^) to obtain the Ru­(bpy)_3_^2+^ transport in nmol·cm^–2^. The transport kinetics of Ru­(bpy)_3_^2+^ versus diffusion time (min) were well described by an exponential decay model fitted using the following “Exp_Xoffset” built-in function in Igor Pro,

2 $$y=y_{0}+A\;\exp\left\{\frac{-\left(x-x_{0}\right)}{\tau }\right\}$$

where *y*_0_ is an offset (Ru­(bpy)_3_^2+^ transport in nmol·cm^–2^), *A* is the amplitude, *x*_0_ is a horizontal offset (time), and τ is the decay time constant. This fit indicates time-dependent accumulation of the analyte in the permeate compartment, which is consistent with first-order kinetics typically associated with diffusion-limited transport across the membrane. The total Ru­(bpy)_3_^2+^ transport for each condition was calculated as the area under the fitted curve over the 3.5 h diffusion duration (30 min conditioning +3 h analyte diffusion). Practically, we numerically summed the fitted values over this time. Using the same 3.5 h duration for all conditions keeps the comparisons time-matched. This duration is long enough to capture the late-time, decreasing-slope part of the trace, yet short enough to limit artifacts such as evaporation, photobleaching, slow drift, and local concentration buildup near the membrane. This analysis does not require a strict steady state (i.e., a clear plateau in the diffusion traces).

### Membrane Incubation Experiments to Quantify Ru­(bpy)_3_^2+^–Nanochannel Attachment

Before the dipping (or incubation) experiments, the membranes were cut into equal (10 mm diameter) circular shapes by using a custom-built hydraulic press. A minimum of three circular samples was tested for each incubation test. Then, the cut membranes were incubated for 1 h in a Ru­(bpy)_3_^2+^ (5 μM)/PBS (1X) solution at different pH. After the analyte staining, the membranes were washed in a pristine PBS solution with a fixed pH (similar to the Ru­(bpy)_3_^2+^ solution pH) either for 5 s (*fast washing*) or 1 h (*long washing*). After this final washing, samples were dried either under high-vacuum conditions for at least 1 h or in a fume hood for a minimum of 24 h. Both approaches yielded consistent fluorescence signals (*n* ≥ 3), confirming their suitability for sufficient water removal from the nanochannels. As detailed in the Supporting Information (Figure S9 and Table S1), we further validated this by testing vacuum drying durations of up to 1 week and observing no significant changes in red-channel intensity or fluorescence lifetime. The dry membranes were then placed under the continuous illumination of a 365 nm light by an Alonfire 3W torch used as a light excitation source. The torch was positioned at a fixed height over a dark surface and plugged into the power outlet to avoid power drops or fluctuations. The photos were acquired with a Thorlabs Camera (DCC1645C – USB 2.0 CMOS Camera) mounted with an objective HR f1.4/22 mm. A 530 nm cutoff filter was placed in front of the camera objective while acquiring the photos. Photos were acquired with the same camera parameters (7 FPS, 140 ms exposure time). They were analyzed with an image processing software, ImageJ, after splitting the color channels and evaluating the mean intensity of the red channel within a circular region of interest (ROI). The average red color intensity values were averaged from a minimum of three replicates of a specific sample, after normalization to the mean red color intensity collected from the nonstained (reference) sample.

### Confocal Microscopy Imaging

After 1 h incubation in Ru­(bpy)_3_^2+^ solution (5 μM) and fast washing and overnight drying, the large nanochannel membranes were imaged by a confocal microscope (Nikon Ti2 Inverted microscope with A1R HD laser-scanning) at 50% RH, 25 °C (lab conditions). The images were taken using a 100× magnification oil immersion objective (NA 1.45), with a pinhole diameter of 1.0 AU and a zoom value of 5×. The excitation laser was set at 489 nm with 100% of its power. The detection was performed in the 595/50 nm channel with a detector gain (PMT HV) of 158. The size of the recorded images is 512 × 512, and the intensity was averaged 8×. The resulting images were analyzed/prepared by image processing software, ImageJ.

## Results and Discussion

pH-dependent nanopore transport dynamics of Ru­(bpy)_3_^2+^ analyte was studied for two different polycarbonate ion-track-etched membranes: (i) large nanochannel membrane (*LNC membrane*) and (ii) small nanochannel membrane (*SNC membrane*) (Figure 1a,b). LNC membrane has an average pore size of 375 nm pore diameter and a pore density of 10^8^ pores/cm^2^. SNC membrane has 15 nm pore diameter and 6 × 10^8^ pores/cm^2^ of pore density (Figure 1a,b and Table 1). The nanochannel diameters of these two membranes are both beyond the EDL thickness, yet the SNC membrane is just at the edge of the regime in which EDL can play a role, while in LNC membranes, the nanochannels are ca. 100 times larger than the EDL, far beyond this regime.^22,45^ As presented in Figure 1a, the roughnesses of the two faces of our PC membranes are different. However, our SEM image analysis (Table 1) and static contact angle (CA_static_) measurements showed that both faces of our membranes have similar nanopore size and liquid/solid interactions (CA_static_), respectively (Figure 1c). In general, all of the faces of the membranes are hydrophilic; thus, their nanochannel wetting is easy (CA_static_ < 90°). One distinctive feature of these membranes is that the aspect ratio, *AR*, of the nanochannels is extremely high (nanochannel length is 9.4 ± 0.5 μm for LNC membranes (*AR* ≈ 23) and 6.5 ± 0.6 μm for SNC membranes (*AR* ≈ 430)), with straight and uniform cross section across the whole thickness of membranes (Figure 1a-left,b).

To study the time-dependent ionic nanochannel diffusion dynamics of Ru­(bpy)_3_^2+^ dye through the membranes at different pH, we used the setup presented in Figure 1d (details in the Experimental Section). The environmental pH (of feed, permeate, and circulating solutions) was fixed to pH 2.5 or pH 7. As presented in Figure S1, the membrane film surface and nanochannels did not suffer from prolonged (3.5 h) contact with buffer solutions at these pH conditions. After 30 min of conditioning, when the membrane was in contact with pristine PBS buffer (1×, pH 2.5 or pH 7), the Ru­(bpy)_3_^2+^ dye was added only inside the feed container (initial concentration of 5 μM) to initiate the osmotic pressure-driven ionic analyte diffusion. 30 min of conditioning with PBS was sufficient to wet and activate all nanochannels. Indeed, more biased wetting of the LNC by ethanol conditioning did not vary significantly the detected time-dependent Ru­(bpy)_3_^2+^ (nmol/cm^2^) permeation compared to those conditioned by PBS solution (Figure S2). The time-dependent diffusion of the Ru­(bpy)_3_^2+^ dyes to the permeate container was quantified by collecting the emission intensity of the analyte (λ_em_ = 608 nm, Figure S3) throughout the entire (3.5 h) diffusion experiment (Figure 1d). As shown in Table 1, the total nanopore opening in this diffusion area (number of nanopores in 0.502 cm^2^ area × single nanopore area) for the LNC membrane is ca. 100 times larger than the total opening of the SNC membrane. To compare the time and pH-dependent diffusion of the cationic analyte through our LNC and SNC membranes, the collected fluorescence emission intensity from the analyte was normalized to the total membrane area in contact with the analyte solution (0.502 cm^2^) (see Figure 2-inset).

**Figure 2:**
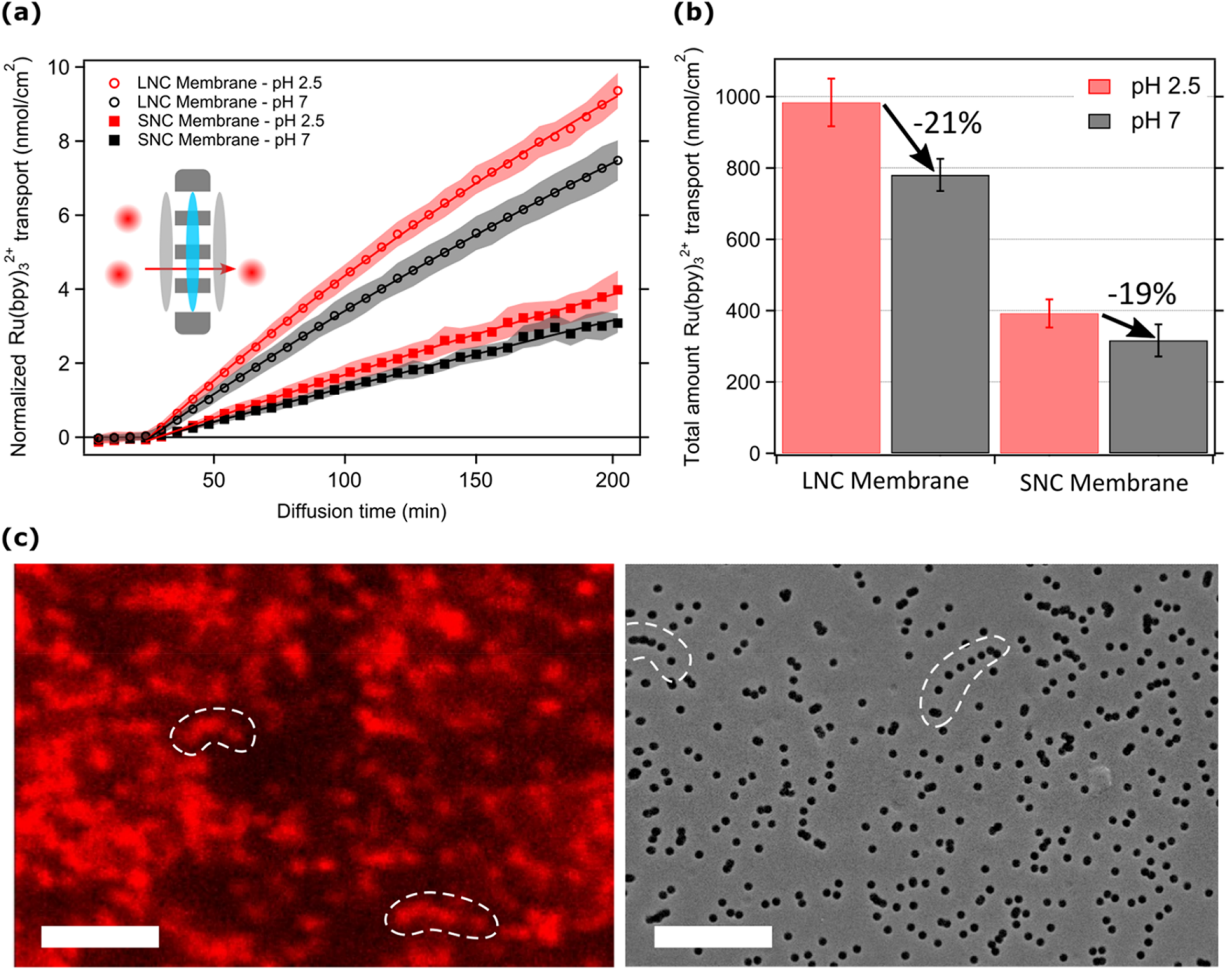
(a) Normalized time-dependent Ru­(bpy)_3_^2+^ (nmol/cm^2^) permeation through the nanochannels of large nanochannel (*LNC*, circles) and small nanochannel (*SNC*, filled squares) membranes at low (red, pH 2.5) and high (black, pH 7) pH. The inset illustrates the area (blue) used for fluorescent intensity normalization, and the gray colored area (same size as the blue one) represents the nanochannel where Ru­(bpy)_3_^2+^ ions diffuse from feed to permeate (red arrow). (b) Bar graphs presenting the normalized total amount of analyte (nmol/cm^2^) diffused into the permeate chamber after 3 h of diffusion, as derived from the integrals of the areas under the diffusion curves in part (a). Error bars are SD and calculated from a minimum of three independent diffusion experiments for each specific condition. (c-left) Confocal image of the Large Nanochannel membrane captured after studying the Ru­(bpy)_3_^2+^ (5 μM) nanopore diffusion in 3 h. (c-right) SEM image presenting the permeate side of the same membrane. White dashed areas help the reader to understand a similar (*not identical*) nanochannel sequence detected in confocal and SEM images. Scale bars are 5 μm.

Both absorption and emission spectra of the Ru­(bpy)_3_^2+^ (5 μM) dye solution in PBS collected at pH 2.5 and pH 7 demonstrate that the cationic dye is stable at the employed pH conditions and that peak position and intensity are not affected by changing environmental pH (Figure S3). Furthermore, the emission intensity increases linearly with absorbance (thus, with concentration) in the concentration regime here monitored (<2.5 μM) (Figure S3b). Thus, the only factor that remains that can affect the intensity changes of the λ_em_ = 608 nm peak is the amount of Ru­(bpy)_3_^2+^ dye diffused from the feed to the permeate. In the literature, the flux of analytes to permeate, in general, is quantified after normalizing the amount of dye (nmol) to the membrane area (for our setup, 0.502 cm^2^) (Figure 2a; gray area). Ru­(bpy)_3_^2+^ dye transport to the permeate is more significant through the LNC membranes compared to SNC membranes for both pH conditions (Figure 2a,b), as expected, owing to the much larger channel openings of the LNC membranes than the SNC membranes (see Table 1). As indicated in the literature, during the irradiation and subsequent alkaline etching process, carboxyl and hydroxyl groups are introduced onto the pore walls of polycarbonate membranes.^6,46–48^ These carboxyl groups deprotonate at neutral pH, resulting in a negative surface charge density of approximately 2 mC/m^2^.^49,50^ Although well established, the extent of deprotonation under our buffer conditions may be further clarified in future independent work, for example, via zeta potential profiling versus pH/ionic strength and XPS on differently conditioned membranes. As known from the literature, negatively charged nanochannels, at high (≥5 mM) analyte concentrations, electrostatically attract the oppositely charged analytes toward the nanochannels and eventually enhance the cation transport to the permeate.^8,9,25,26^ Interestingly, we realized that the transport of cationic dyes is more limited inside the negatively charged channels (pH 7) compared to the diffusion in the neutral channels (pH 2.5). Indeed, as presented in Figure 2b, the total amount of Ru­(bpy)_3_^2+^ dye diffused inside the permeate at the end of 3.5 h diffusion time (integral of each corresponding curve in Figure 2a), for both types of membranes, is ca. 21% when the nanochannels are negatively charged. It is important to highlight that the factor limiting the cationic transport at low analyte concentrations acts similarly at both nanochannel sizes: LNC, where diffusion happens in the bulk conductance range (pore diameter ca. 400 nm), and SNC membranes, where the diffusion happens close to the permselective range (pore diameter ca. 15 nm).^22^

At such a low analyte concentration (μM level), the electrostatic interaction between the cationic analyte Ru­(bpy)_3_^2+^ and the deprotonated nanochannel walls becomes prominent and may limit transport when the nanochannels are negatively charged. Indeed, confocal images of the LNC membrane after the Ru­(bpy)_3_^2+^ diffusion test at high pH further confirm that the higher amount of Ru­(bpy)_3_^2+^ dye was attached to the nanochannels at high pH compared to low pH (Figures 2c and S4b): the nanochannels are clearly individually visible, as shown in Figure 2c, which also displays the good matching with nanochannels density and morphology as visible in the SEM image. In particular, confocal images make even the closely packed nanochannels visible (areas presented by a white dashed border). By contrast, the signal from the small nanochannels was too low (and the diameter and average pore-to-pore distance were too small) for confocal imaging of the SNC membranes (Figure S4a). Another key issue to consider is the reversibility of such interactions. As shown in detail by recent FCS studies, reversible analyte-nanopore interactions at very low concentrations result in sequences of attachment and detachment events and, therefore, slow *transient adsorption-mediated diffusion*.^51–53^ To investigate the analyte-wall interactions, we performed various dipping experiments by incubating our membranes inside the 5 μM Ru­(bpy)_3_^2+^ dye solutions (similar to those used as feed solution in Figure 2) at pH 2.5 and pH 7 (Figure 3) for 1 h. After incubation, we washed these stained membranes in pristine PBS solutions (0 mM 5 μM Ru­(bpy)_3_^2+^; pH 2.5 or pH 7) very quickly in 5 s (aiming to remove the dye present in the wetting solution, Figure 3b) or slowly for 1 h (Figure 3c, aiming to remove also the dye reversibly attached on the nanochannel walls, Figure S4).

**Figure 3:**
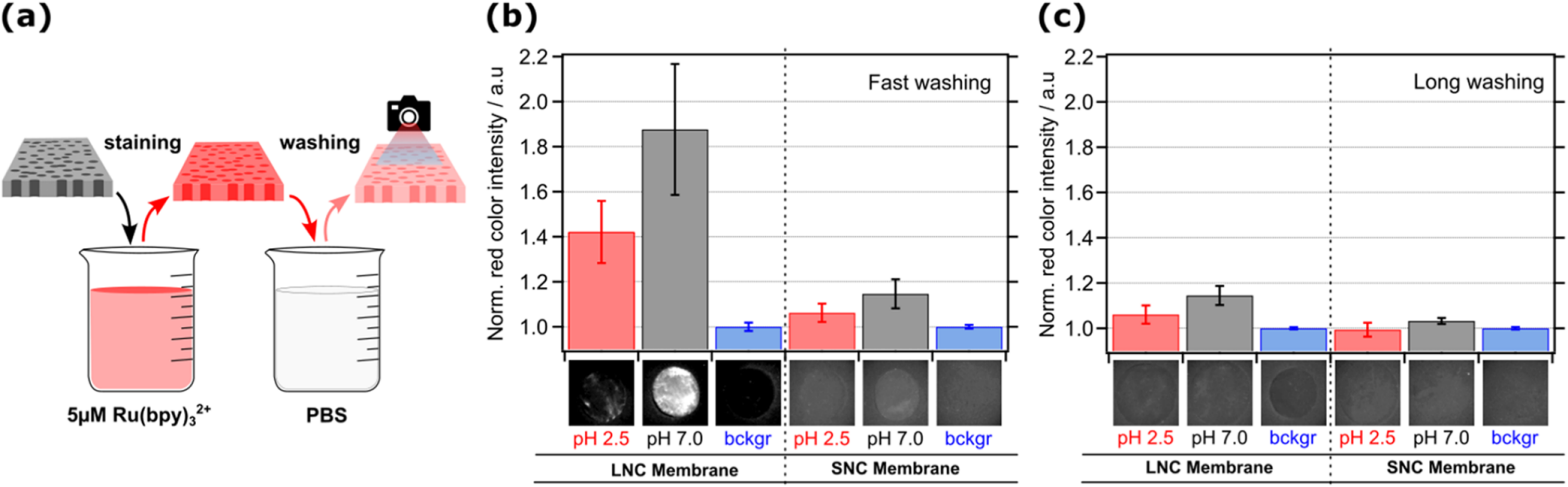
Dipping test—nanochannel staining by Ru­(bpy)_3_^2+^—experiments of membranes. (a) Schematic representation of the dipping experiments, where membranes were first immersed in a 5 μM Ru­(bpy)_3_^2+^ solution (for 1 h) and then washed in only PBS for (b) 5 s (fast washing) or (c) 1 h (long washing). After washing, the samples were left under vacuum for a minimum of 1 h. Finally, the dried samples were illuminated with 365 nm light, and photos were taken using a Thorlabs camera (see Methods for more details). (b, c) Bar graphs presenting the background normalized red light intensities detected from the photographic images (as insets placed under each corresponding bar) of large nanochannel (*LNC*, left) and small nanochannel (*SNC*, right) membranes after their dipping tests at different pH. Error bars in all of the bar graphs are SD from three independent dipping experiments.

Photographic images in the inset of Figure 3b,c were taken under the illumination of 365 nm light, and the emission was collected above the cutoff wavelength of 530 nm (see Methods for details). Bar graphs in Figure 3b,c present the normalized image analysis of the red color intensity calculated from these photos (see Methods). Both the photos and the bar graph results show that at high and low pH levels, more Ru­(bpy)_3_^2+^ dye remains in the larger area pore walls of the LNC membrane compared to the SNC membrane. For both LNC and SNC membranes, we observe approximately twice the emission intensity after dipping at high pH (pH 7), when the membrane is negatively charged. This confirms that electrostatic interactions play a significant role in the adsorption of the analyte onto the nanochannel walls. Interestingly, at low pH, the red emission is significantly higher than the reference membrane, suggesting that the dye also adsorbs without negatively charged carboxylate functional groups, possibly via hydrophobic interactions between the polymeric nanochannels and the analyte. Membranes washed for a longer time (1 h) to remove the adsorbed Ru­(bpy)_3_^2+^ dye from nanochannels as much as possible show a much lower emission signal (ca. 5 times lower than fast-washed membranes), indicating a substantial reversibility of the interactions leading to adsorption of the dye onto the nanochannel walls. However, after such a long washing process, the emission signal was very low; yet, we could still image isolated nanochannels in LNC membranes with good resolution, as indicated by the diameter measured via confocal imaging, which matched very well with the diameter calculated from SEM images (Figure S4c). These experiments overall show that dynamic attachment and detachment of the analyte (*transient adsorption*) is possible and can be responsible for at least a fraction of the diffusion properties of the membranes (Figure 2). In our system, slower transport of Ru­(bpy)_3_^2+^ detected at a high pH in charged nanochannels can be described as *diffusion mediated by transient surface adsorption*. Here, ‘transient adsorption’ refers to reversible analyte binding and release at the channel surface, which slows the effective diffusion rate but does not imply lateral migration along the wall (i.e., not true surface diffusion). Our rationale for adopting the transient adsorption framework is that the nanochannel diameter of our membranes largely exceeds the Debye length (λ_D_ ∼ 0.8 nm in 1× PBS), making discrete site-to-site hopping unlikely.^54–56^ Diffusion, upon reversible adsorption–desorption events of Ru­(bpy)_3_^2+^, may be predominantly confined within the electric double layer (EDL) region where electrostatic attraction is strongest.^50,57,58^ This framework results in transient adsorption-mediated diffusion that slows overall mobility, consistent with experimental observations and with continuum electrokinetic models of ion diffusion near charged interfaces.^59^ Although both LNC and SNC pore diameters exceed the Debye length in 1× PBS, the 15 nm SNC lies closer to the EDL-interaction regime. We therefore cannot exclude a modest electrostatic selectivity in SNC; however, because 2λ_D_ (∼1.6 nm) ≪ *d* (SNC: 15 nm; LNC: 400 nm) and the pH-dependent attenuation is similar to LNC (≈21 vs ≈19%), we interpret the deviations primarily as transient adsorption-mediated effects rather than strong permselectivity under our conditions.

Besides, to relate the diffusion data from our experiments to classical permeation parameters, we map the characteristic time constant (τ; τ_pH 2.5_ = 388.3; τ_pH 7_ = 535.1) obtained from our single-exponential fits (eq 2) to permeability (*P*) using the equal-volume side-by-side model.^60–62^ In this well-stirred framework, the donor–receiver exchange is first-order with τ = *V*/(2*PA*) (*V*: chamber volume [m^3^], *A*: membrane area [m^2^], *P*: permeability [m·s^–1^]; see the SI for details). With the identical geometry of our experimental setup, τ∝1/*P*; therefore,

$$\frac{P_{\mathrm{pH}2.5}}{P_{\mathrm{pH}7}}=\frac{\tau_{\mathrm{pH}7}}{\tau_{\mathrm{pH}2.5}}=\frac{535.1}{388.3}\approx 1.38$$

indicating ∼38% higher permeability at low pH (pH 2.5), where transient wall–analyte interactions are suppressed. Conversely, the larger τ at pH 7 reflects interaction-induced retardation that decreases the effective permeability.

Raising the pH has, therefore, emerged as a simple way to decrease the diffusion through nanochannels, owing to the role of electrostatic interactions that can be “switched” on and off based on pH. Electrostatic interactions are expected to be very sensitive to the ionic strength of the solution: we therefore tested the fundamental role of ionic strength at the charged nanochannel walls and thus on the transient adsorption mechanism triggered cation transport, by measuring Ru­(bpy)_3_^2+^ diffusion in pure water (without PBS) (Figure S5). From the diffusion tests performed at low pH when the nanochannel walls are neutral, we collected a similar total amount of diffused Ru­(bpy)_3_^2+^ from both systems with pure water ([HCl] ≈ 3.2 mM) and PBS. However, when the nanochannel walls are deprotonated (pH ≈ 7, pure water), the Ru­(bpy)_3_^2+^ diffusion decreases much more—45% less compared to the diffusion at low pH—in pure water, compared to what was measured in PBS (−21%). This expected behavior, due to partial screening of electrostatic interactions, for example, by monovalent Na^+^, further proves that the observed decrease in diffusion rate is ascribable to the electrostatic interactions taking place between analyte and nanochannel walls, and as such, the diffusion rate can be finely adjusted by tuning the strength of electrostatic interactions, i.e., (i) by protonating the negative sites at nanochannels walls or (ii) by increasing the ionic strength and screening the electrostatic charges.^52,63–65^

Besides these two possibilities, we tested the effect of adding a divalent cation that can also strongly bind to carboxylate groups and, therefore, more efficiently compete with the analyte for electrostatic interactions with the nanochannel walls. At the conditioning stage, different amounts of CaCl_2_ were used to gradually neutralize the negatively charged sites of Large nanochannels. Similar to the experiment performed for Figure 2, Ru­(bpy)_3_^2+^ dye was added to the system after the conditioning. Changes in the Ru­(bpy)_3_^2+^ analyte transport in the presence of the competitor dication Ca^2+^ were then studied by collecting time-dependent fluorescent signal change, exactly as previously done in the absence of Ca^2+^ (Figure 4a). The black and red-filled diamonds in Figure 4a represent the transport behavior already presented in Figure 2a, where they represent the transport in conditions of maximum electrostatic interactions, resulting in the largest transient adsorption between the analyte and nanochannel walls (pH 7), and conditions of minimum electrostatic interactions (faster diffusion at pH 2.5). Interestingly, we found that, upon increasing the concentration of the competitor Ca^2+^ at pH 7, the electrostatic interactions of the dye at nanochannels are decreased, and the transient adsorption-mediated diffusion mechanism is progressively “quenched.” As a result, the diffusion rate smoothly increases from the one observed at pH 7 in the absence of Ca^2+^ (maximal electrostatic interaction and transient adsorption) to the one observed at pH 2.5 (no electrostatic interactions and minimal analyte–nanochannel wall interaction) (Figure 4). This gradual increase in cation transport approaches a plateau between 7 and 20 mM Ca^2+^; no further rise is observed at 50 mM Ca^2+^, and the 0 mM Ca^2+^, pH 2.5 measurement—used as a neutral channel reference—represents the highest expected Ru­(bpy)_3_^2+^ transport among the conditions. As also reported in the literature, CaCl_2_ concentrations up to 50 mM do not significantly affect solution viscosity;^66^ thus, the observed plateau in Ru­(bpy)_3_^2+^ transport arises from the saturation of negatively charged surface sites by excess Ca^2+^ ions, rather than changes in bulk fluid properties (see Figures 4 and S6b).

**Figure 4:**
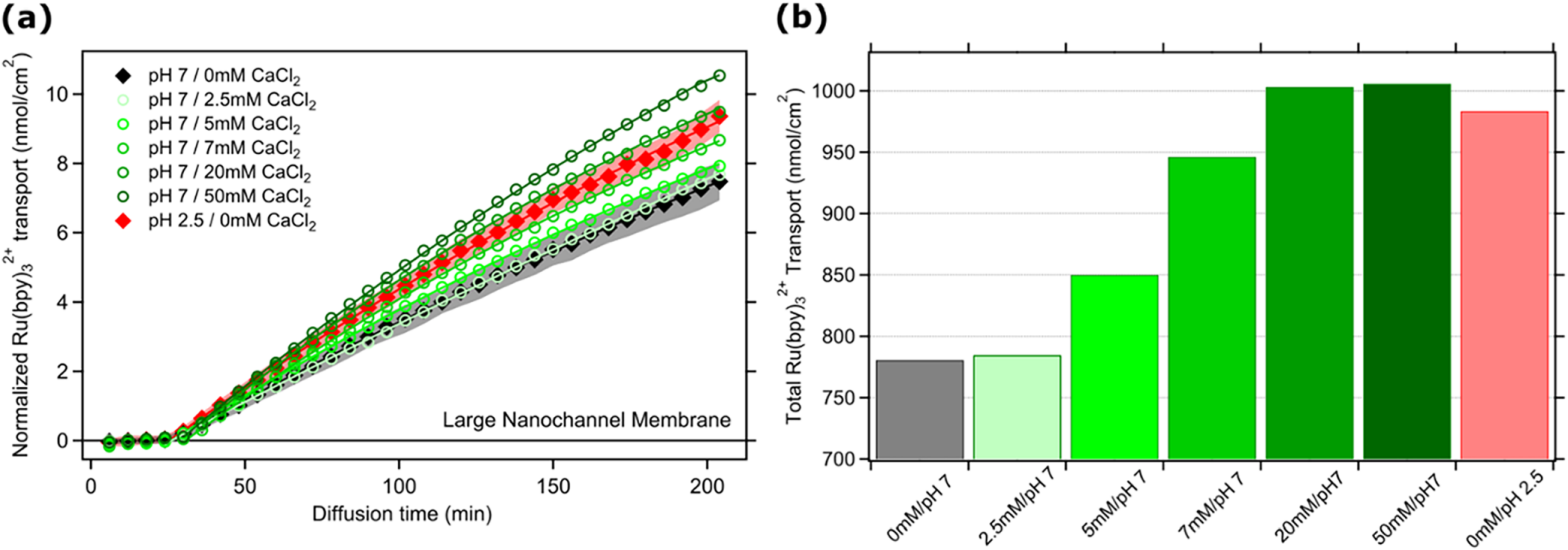
(a) Normalized time-dependent Ru­(bpy)_3_^2+^ (nmol/cm^2^) transport to the permeate chamber through large nanochannels at changing pH and added CaCl_2_ (called *cocation*) with different amounts at alkaline pH. Straight lines inside the same color markers represent the exponential fittings to the collected data. Error bars are SD from at least three measurements from each specific condition. (b) Bar graphs presenting the total transported Ru­(bpy)_3_^2+^ through the membrane at different pH and CaCl_2_ concentration conditions after 3.5 h.

To evaluate the thermodynamic parameters of adsorption of charged analytes onto deprotonated nanochannel surfaces, we conducted two complementary experiments (Figure S6; see SI): (1) the adsorption trend of Ru­(bpy)_3_^2+^ was obtained by dipping LNC membranes into Ru­(bpy)_3_^2+^ solutions of increasing concentrations (0–500 μM) at pH = 7, followed by the relative quantification of adsorbed Ru­(bpy)_3_^2+^ via fluorescence imaging as detailed in SI (Figure S6a); (2) total occupied binding sites of deprotonated LNC nanochannels by Ca^2+^ ions that was evaluated by using the total amount of Ru­(bpy)_3_^2+^ (5 μM) diffused through LNC nanochannels in the presence of increasing concentrations of CaCl_2_ (2.5–50 mM, Figure 4b) (Figure S6b). In both cases, Langmuir isotherm fitting was applied to obtain dissociation constants (*K*_D_), yielding values of 3.94 μM for Ru­(bpy)_3_^2+^ and 10.45 mM for Ca^2+^. The significantly weaker affinity of Ca^2+^ justifies the high excess of Ca^2+^ (≥500-fold relative to Ru­(bpy)_3_^2+^) needed to partially neutralize the surface charge and substantially enhance Ru­(bpy)_3_^2+^ transport, approaching the levels observed under acidic pH conditions, where the nanochannels are noncharged and electrostatic interactions are minimized (see Figure 4b). Beyond electrostatics, π–π contacts, hydrogen bonding, and hydrophobic association of Ru­(bpy)_3_^2+^ with the polycarbonate matrix likely contribute to adsorption (consistent with its reversible attachment at low pH; see Figure 3b, red bars), so the reported Langmuir parameters should be interpreted as effective affinities that encompass both electrostatic and specific interactions. Finally, a similar enhancement of Ru­(bpy)_3_^2+^ transport at high pH and addition of competing ions was also observed upon the addition of monovalent ions. As shown in Figures S5 and S7, increasing the NaCl concentration from 0 mM (Milli-Q) to 1X PBS (∼137 mM NaCl) resulted in greater analyte transport due to electrostatic screening of the negatively charged pore walls. Notably, the effect became more pronounced at 637 mM NaCl, where the Ru­(bpy)_3_^2+^ transport approached values observed in neutral nanochannels (pH 2.5) (Figure S7). These results confirm that both mono- and divalent ions can modulate surface interactions through ionic screening, although divalent ions are more efficient on a per-ion basis.

The hereby discussed experimental findings are gathered and summarized with illustrations in Figure 5. Time-dependent Ru­(bpy)_3_^2+^ dye permeation results show that at micromolar (5 μM) analyte concentrations and at higher pH (pH 7), when the nanopores are deprotonated (negatively charged), the analyte transport is more limited for both nanochannels with large (400 nm) and small (15 nm) nanopore diameters (Figure 2). Photographic images of our Ru­(bpy)_3_^2+^ dye-stained membranes and their corresponding confocal images (Figures 2c, 3, S4, and S8) demonstrate that such limited transport at high pH is due to cationic analyte attachment at nanochannel walls at high pH. Moreover, these experiments also clearly indicated that the Ru­(bpy)_3_^2+^ dye – negatively charged nanopore interaction is reversible. In other words, the Ru­(bpy)_3_^2+^ dyes during our diffusion tests at low concentrations attach and detach throughout the diffusion test during 3 h (red bent arrows in Figure 5). However, such electrostatic attraction between cationic analyte and pore walls at high pH generally results in a larger amount of Ru­(bpy)_3_^2+^ dye present at the nanochannels than those quantified at low pH. Contrarily, to date, similar time-dependent nanopore transport studies, in general, were performed at significantly higher analyte concentrations (a couple of mM). Interestingly, their results showed the opposite trend to our findings: the larger the pH, the larger the cation transport to the permeate. However, this transport behavior has also been explained by our explanations: a more pronounced electrostatic interaction between the cationic analytes and the pore walls. Beyond these studies, our findings indicate for the first time that at very low concentrations (μM), this electrostatic interaction, and thus the reversible attachment–detachment mechanism of the analyte, dominate the overall transport behavior and limit the dye from diffusing into the permeate. Compared to the nanochannel activities at high pH, cationic analytes do not interact significantly with the neutral nanopores when the membrane is in low pH conditions; thus, Ru­(bpy)_3_^2+^ dye transport occurs faster at low pH. However, our further photophysical characterization studies also confirm that even at low pH, there is still (although more limited compared to high pH ones) analyte–nanochannel interaction, probably occurring via a hydrophobic interaction between the neutral nanochannel walls and the cationic analyte. In our upcoming studies, we will systematically and in more detail investigate the concentration and analyte–pore wall interaction effects in similar nanochannels.

**Figure 5:**
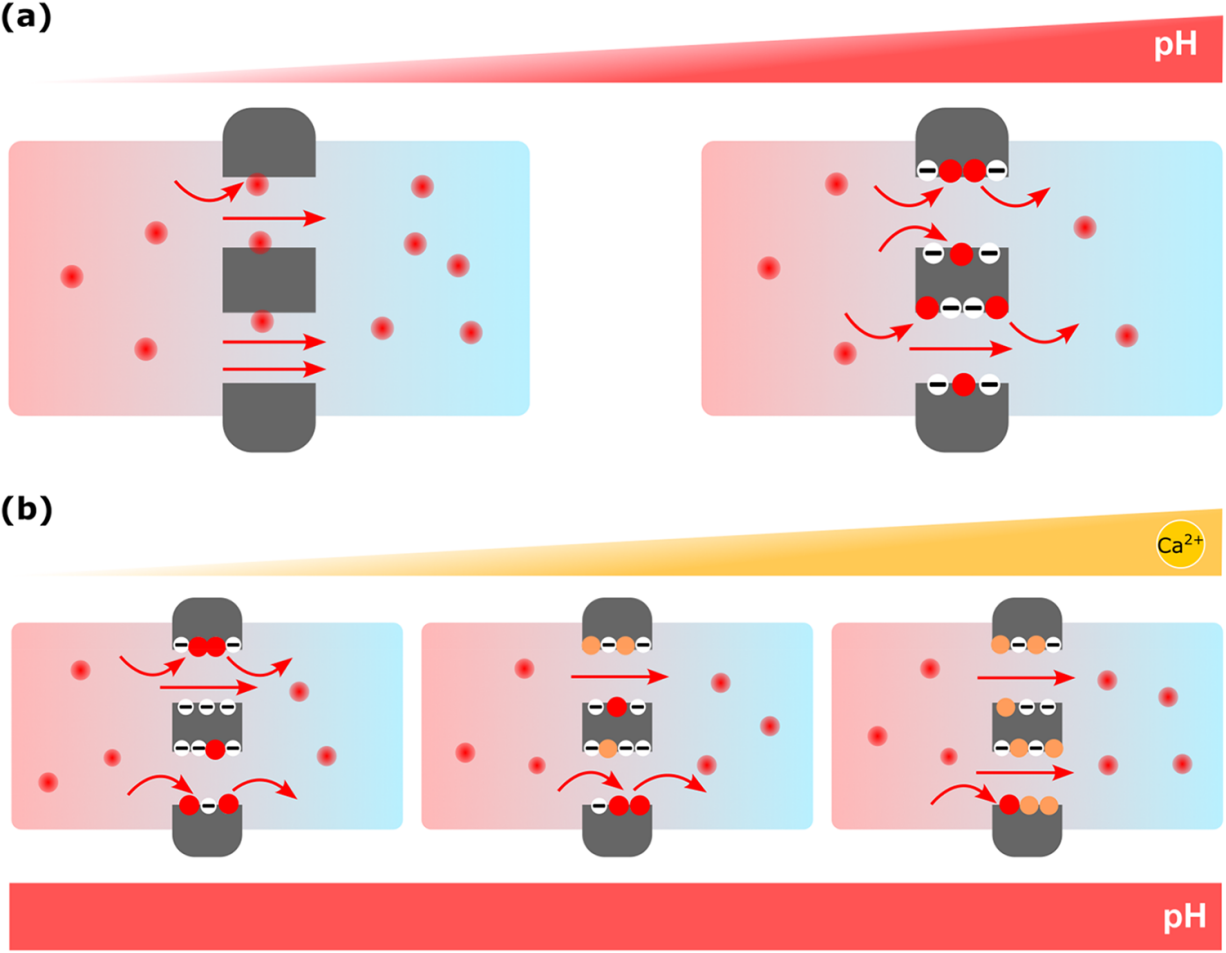
(a) Illustration presenting how Ru­(bpy)_3_^2+^ dye–nanochannel interactions at high pH and micrometer-scale analyte concentrations regulate the nanochannel transport of cationic analytes. Illustrations in part (b) present how a higher amount of divalent competitor cation, CaCl_2_ addition, neutralizes the nanochannels at high pH conditions and how such CaCl_2_ binding limits the cationic analyte–nanochannel interactions and, thus, enhances the analyte diffusion. Red and orange balls represent the Ru­(bpy)_3_^2+^ dye and Ca^2+^ ions, respectively.

Second, we could fine-tune the Ru­(bpy)_3_^2+^ transport at high pH by gradually adding a competitor divalent cation, Ca^2+^ (Figure 4), as well as monovalent ions (Na^+^ and Cl^–^, Figures S5 and S7) to the system (Figure 5b). Basically, under high pH conditions and during the conditioning stage (prior to the Ru­(bpy)_3_^2+^ addition), the addition of Ca^2+^ ions could gradually neutralize (occupy) the deprotonated COO^–^ sites at the nanochannels. Therefore, being negatively charged sites already occupied (neutralized) by Ca^2+^ ions, Ru­(bpy)_3_^2+^ could diffuse faster to the feed. In the end, adding Ca^2+^ ions alone at high pH can tune both analyte–pore wall interactions in polymeric nanochannels, thereby enhancing the transport of cationic analyte to the permeate to the same level as diffusion in neutral nanochannels. With good agreement with our previous study, which explained the role of Ca^2+^-binding on the transport at mesoporous silica nanopores,^35^ the larger amount of Ca^2+^ ions could gradually neutralize the nanopores up to a particular Ca^2+^ ion concentration (between 7 and 20 mM for our large nanochannel membranes). Beyond this saturation level, where nanochannels are almost fully neutralized, a larger neutralization via more Ca^2+^ ion addition is simply not possible, and the cationic analyte transport remains at a similar level to neutral nanochannels.

## Conclusions

This study provides new insights into the fundamental interactions between cationic analytes and nanoporous membranes at low concentrations, highlighting the role of electrostatic interactions in transport dynamics. Compared to previous studies, it bridges bulk measurements performed on electroactive analytes^7,23,24^ at millimolar concentrations with highly specialized investigations performed at individual nanopores with single-molecule sensitive methods such as FCS or single-molecule microscopy.^2,29,51,67^ Interestingly, this study employs a simple and cost-effective setup based on a spectrophotometer or spectrofluorometer to monitor the diffusion of submicromolar concentrations of photoactive species.

Our findings reveal that negatively charged nanochannels hinder Ru­(bpy)_3_^2+^ diffusion, a behavior attributed to reversible analyte attachment and detachment along the pore walls. This observed behavior contrasts with trends observed at higher analyte concentrations,^8,9,25,26^ while it finds confirmation in FCS investigations performed at nanomolar concentration,^51,52,68^ underscoring the reverse relationship between analyte–pore wall interactions and concentration regimes.

In addition, we demonstrate that these interactions can be switched on and off by simply adjusting the pH: the analyte flux can be reversibly reduced by ≈20% by changing the pH from acidic to neutral and vice versa.

Furthermore, our simple approach allows us to demonstrate that the net flux can be finely tuned not only by adjusting the pH but also by using a divalent competitor cation (calcium ions), which effectively neutralizes negatively charged nanopores at high pH, enhancing diffusion to levels observed in acidic environments. On the contrary, a decrease in the ionic strength maximizes the effect of electrostatic interactions and minimizes the transport of cationic analytes.

This novel approach offers a promising strategy for regulating mass transport in nanoporous systems with implications for various applications, including selective labeling and targeted molecular separation.

These findings contribute to a broader understanding of nanopore–analyte interactions, laying the groundwork for future studies to explore concentration-dependent behaviors and other environmental factors. By bridging gaps in our knowledge of low-concentration transport mechanisms, this work advances the design of nanoporous materials for next-generation microfluidic and sensing technologies.

## Supplementary Material



## Notes

Supporting Information methods (steady-state UV–vis and fluorescence spectroscopy; calibration curves for Ru­(bpy)_3_^2+^ quantification; ethanol conditioning; automated diffusion with Milli-Q and NaCl; confocal imaging; derivation of fitted time constant (τ, from exponential diffusion fits)–permeability relation; Ca^2+^ site occupancy quantification; Langmuir isotherm fittings; vacuum drying experiments; fluorescence lifetime decays); Supporting Information figures (SEM images of membranes after diffusion at different pH; Ru­(bpy)_3_^2+^ diffusion after ethanol conditioning; UV–vis and fluorescence calibration spectra; 2D and 3D confocal images at different pH; diffusion with Milli-Q vs PBS; red-signal vs Ru­(bpy)_3_^2+^ concentration; binding site occupancy vs Ca^2+^ concentration; diffusion in the presence of NaCl; non-normalized red-channel intensities; lifetime analysis at different drying durations; Figures S1–S9); and Supporting Information table of fluorescence lifetimes (Table S1) (PDF)

## References

[1] Zhang H., Tian Y., Jiang L. (2016). Fundamental Studies and Practical
Applications of Bio-Inspired Smart Solid-State Nanopores and Nanochannels. Nano Today.

[2] Varol H. S., Kaya D., Contini E., Gualandi C., Genovese D. (2024). Fluorescence
Methods to Probe Mass Transport and Sensing in Solid-State Nanoporous
Membranes. Mater. Adv..

[3] Liu H., Zhou Q., Wang W., Fang F., Zhang J. (2023). Solid-State
Nanopore Array: Manufacturing and Applications. Small.

[4] He Y., Tsutsui M., Zhou Y., Miao X. S. (2021). Solid-State Nanopore
Systems: From Materials to Applications. NPG
Asia Mater..

[5] Pérez-Mitta G., Albesa A. G., Trautmann C., Toimil-Molares M. E., Azzaroni O. (2017). Bioinspired Integrated Nanosystems
Based on Solid-State
Nanopores: “Iontronic” Transduction of Biological, Chemical
and Physical Stimuli. Chem. Sci..

[6] Ma T., Janot J., Balme S. (2020). Track-Etched
Nanopore/Membrane: From
Fundamental to Applications. Small Methods.

[7] Laucirica G., Toum Terrones Y., Cayón V., Cortez M. L., Toimil-Molares M. E., Trautmann C., Marmisollé W., Azzaroni O. (2021). Biomimetic Solid-State
Nanochannels for Chemical and Biological Sensing Applications. Trends Anal. Chem..

[8] Nguyen Q. H., Ali M., Bayer V., Neumann R., Ensinger W. (2010). Charge-Selective Transport
of Organic and Protein Analytes through Synthetic Nanochannels. Nanotechnology.

[9] Nguyen Q. H., Ali M., Nasir S., Ensinger W. (2015). Transport Properties of Track-Etched
Membranes Having Variable Effective Pore-Lengths. Nanotechnology.

[10] Wen Q., Yan D., Liu F., Wang M., Ling Y., Wang P., Kluth P., Schauries D., Trautmann C., Apel P., Guo W., Xiao G., Liu J., Xue J., Wang Y. (2016). Highly Selective
Ionic Transport through Subnanometer
Pores in Polymer Films. Adv. Funct Mater..

[11] Ali M., Yameen B., Cervera J., Ramírez P., Neumann R., Ensinger W., Knoll W., Azzaroni O. (2010). Layer-by-Layer
Assembly of Polyelectrolytes into Ionic Current Rectifying Solid-State
Nanopores: Insights from Theory and Experiment. J. Am. Chem. Soc..

[12] Zhang S., Xia F., Demoustier-Champagne S., Jonas A. M. (2021). Layer-by-Layer Assembly
in Nanochannels: Assembly Mechanism and Applications. Nanoscale.

[13] Lu J., Zhang H., Hou J., Li X., Hu X., Hu Y., Easton C. D., Li Q., Sun C., Thornton A. W., Hill M. R., Zhang X., Jiang G., Liu J. Z., Hill A. J., Freeman B. D., Jiang L., Wang H. (2020). Efficient
Metal Ion Sieving in Rectifying Subnanochannels Enabled by Metal–Organic
Frameworks. Nat. Mater..

[14] Cayón V. M., Laucirica G., Toum Terrones Y., Cortez M. L., Pérez-Mitta G., Shen J., Hess C., Toimil-Molares M. E., Trautmann C., Marmisollé W.
A., Azzaroni O. (2021). Borate-Driven
Ionic Rectifiers Based on Sugar-Bearing Single Nanochannels. Nanoscale.

[15] Ulrich N., Spende A., Burr L., Sobel N., Schubert I., Hess C., Trautmann C., Toimil-molares M. E. (2021). Conical
Nanotubes Synthesized by Atomic Layer Deposition of Al2O3, TiO2, and
SiO2 in Etched Ion-Track Nanochannels. Nanomaterials.

[16] Usman M., Ali M., Al-Maythalony B. A., Ghanem A. S., Saadi O. W., Ali M., Jafar Mazumder M. A., Abdel-Azeim S., Habib M. A., Yamani Z. H., Ensinger W., Al-Maythalony B. A. (2020). Highly
Efficient Permeation and Separation of Gases with Metal-Organic Frameworks
Confined in Polymeric Nanochannels. ACS Appl.
Mater. Interfaces.

[17] Yameen B., Ali M., Álvarez M., Neumann R., Ensinger W., Knoll W., Azzaroni O. (2010). A Facile Route for the Preparation
of Azide-Terminated Polymers. “Clicking” Polyelectrolyte
Brushes on Planar Surfaces and Nanochannels. Polym. Chem..

[18] Yameen B., Ali M., Neumann R., Ensinger W., Knoll W., Azzaroni O. (2009). Synthetic
Proton-Gated Ion Channels via Single Solid-State Nanochannels Modified
with Responsive Polymer Brushes. Nano Lett..

[19] Wu Y., Qian Y., Niu B., Chen J., He X., Yang L., Kong X. Y., Zhao Y., Lin X., Zhou T., Jiang L., Wen L. (2021). Surface Charge Regulated
Asymmetric Ion Transport in Nanoconfined Space. Small.

[20] Zhang Z., Sui X., Li P., Xie G., Kong X. Y., Xiao K., Gao L., Wen L., Jiang L. (2017). Ultrathin and Ion-Selective Janus
Membranes for High-Performance Osmotic Energy Conversion. J. Am. Chem. Soc..

[21] Varol H. S., Herberger T., Kirsch M., Mikolei J., Veith L., Kannan-Sampathkumar V., Brand R. D., Synatschke C. V., Weil T., Andrieu-Brunsen A. (2023). Electropolymerization
of Polydopamine
at Electrode-Supported Insulating Mesoporous Films. Chem. Mater..

[22] Faucher S., Aluru N., Bazant M. Z., Blankschtein D., Brozena A. H., Cumings J., Pedro De
Souza J., Elimelech M., Epsztein R., Fourkas J. T., Rajan A. G., Kulik H. J., Levy A., Majumdar A., Martin C., McEldrew M., Misra R. P., Noy A., Pham T. A., Reed M., Schwegler E., Siwy Z., Wang Y., Strano M. (2019). Critical Knowledge
Gaps in Mass Transport through Single-Digit
Nanopores: A Review and Perspective. J. Phys.
Chem. C.

[23] Lu W., Hu R., Tong X., Yu D., Zhao Q. (2020). Electro-Optical
Detection
of Single Molecules Based on Solid-State Nanopores. Small Struct..

[24] Fried J. P., Wu Y., Tilley R. D., Gooding J. J. (2022). Optical Nanopore Sensors for Quantitative
Analysis. Nano Lett..

[25] Duznovic I., Diefenbach M., Ali M., Stein T., Biesalski M., Ensinger W. (2019). Automated Measuring
of Mass Transport through Synthetic
Nanochannels Functionalized with Polyelectrolyte Porous Networks. J. Membr. Sci..

[26] Yang Q., Lin X., Su B. (2016). Molecular Filtration
by Ultrathin and Highly Porous
Silica Nanochannel Membranes: Permeability and Selectivity. Anal. Chem..

[27] Van
Der Heyden F. H. J., Bonthuis D. J., Stein D., Meyer C., Dekker C. (2007). Power Generation by Pressure-Driven Transport of Ions
in Nanofluidic Channels. Nano Lett..

[28] Kumarasinghe R., Ito T., Higgins D. A. (2020). Nanoconfinement
and Mass Transport in Silica Mesopores:
The Role of Charge at the Single Molecule and Single Pore Levels. Anal. Chem..

[29] Dong B., Mansour N., Huang T. X., Huang W., Fang N. (2021). Single Molecule
Fluorescence Imaging of Nanoconfinement in Porous Materials. Chem. Soc. Rev..

[30] Wu Y., Gooding J. J. (2022). The Application
of Single Molecule Nanopore Sensing
for Quantitative Analysis. Chem. Soc. Rev..

[31] Shendure J., Balasubramanian S., Church G. M., Gilbert W., Rogers J., Schloss J. A., Waterston R. H. (2017). DNA Sequencing at 40: Past, Present
and Future. Nature.

[32] Gilboa T., Garden P. M., Cohen L. (2020). Single-Molecule
Analysis of Nucleic
Acid Biomarkers – A Review. Anal. Chim.
Acta.

[33] Wang P., Wang M., Liu F., Ding S., Wang X., Du G., Liu J., Apel P., Kluth P., Trautmann C., Wang Y. (2018). Ultrafast Ion Sieving Using Nanoporous Polymeric Membranes. Nat. Commun..

[34] Xin W., Jiang L., Wen L. (2022). Engineering Bio-Inspired Self-Assembled
Nanochannels for Smart Ion Transport. Angew.
Chem., Int. Ed..

[35] Varol H. S., Förster C., Andrieu-Brunsen A. (2023). Ligand-Binding Mediated Gradual Ionic
Transport in Nanopores. Adv. Mater. Interfaces.

[36] Ali M., Nasir S., Ramirez P., Cervera J., Mafe S., Ensinger W. (2012). Calcium Binding and
Ionic Conduction in Single Conical
Nanopores with Polyacid Chains: Model and Experiments. ACS Nano.

[37] Bashford C. L. (1995). Membrane
Pores-From Biology to Track-Etched Membranes. Biosci. Rep..

[38] Lu C., Hu C., Ritt C. L., Hua X., Sun J., Xia H., Liu Y., Li D. W., Ma B., Elimelech M., Qu J. (2021). In Situ Characterization of Dehydration during Ion Transport in Polymeric
Nanochannels. J. Am. Chem. Soc..

[39] Kim S., Choi H., Kim B., Lim G., Kim T., Lee M., Ra H., Yeom J., Kim M., Kim E., Hwang J., Lee J. S., Shim W. (2023). Extreme Ion-Transport
Inorganic 2D Membranes for Nanofluidic Applications. Adv. Mater..

[40] Xin W., Fu J., Qian Y., Fu L., Kong X. Y., Ben T., Jiang L., Wen L. (2022). Biomimetic
KcsA Channels with Ultra-Selective
K+ Transport for Monovalent Ion Sieving. Nat.
Commun..

[41] Karge, H. G. ; Weitkamp, J. Molecular Sieves: Adsorption and Diffusion; Karge, H. G. ; Weitkamp, J. , Eds.; Berlin, 2008; Vol. 7.

[42] Guo Z., Zhang Y., Dong Y., Li J., Li S., Shao P., Feng X., Wang B. (2019). Fast Ion Transport
Pathway Provided by Polyethylene Glycol Confined in Covalent Organic
Frameworks. J. Am. Chem. Soc..

[43] Varol H. S., Srivastava A., Kumar S., Bonn M., Meng F., Parekh S. H. (2020). Bridging Chains Mediate Nonlinear Mechanics of Polymer
Nanocomposites under Cyclic Deformation. Polymer
(Guildf).

[44] Varol H. S., Seeger S. (2022). Fluorescent Staining of Silicone Micro- and Nanopatterns
for Their Optical Imaging. Langmuir.

[45] Pérez-Mitta G., Toimil-Molares M. E., Trautmann C., Marmisollé W.
A., Azzaroni O. (2019). Molecular
Design of Solid-State Nanopores: Fundamental
Concepts and Applications. Adv. Mater..

[46] Keesom W. H., Zelenka R. L., Radke C. J. (1988). A Zeta-Potential Model for Ionic
Surfactant Adsorption on an Ionogenic Hydrophobic Surface. J. Colloid Interface Sci..

[47] Fleischer, R. L. ; Price, P. B. ; Walker, R. M. Nuclear Tracks in Solids: Principles and Applications. 2022. 10.1038/scientificamerican0669-30 5769561

[48] Paoli R., Bulwan M., Castaño O., Engel E., Rodriguez-Cabello J.
C., Homs-Corbera A., Samitier J. (2020). Layer-by-Layer Modification Effects
on a Nanopore’s Inner Surface of Polycarbonate Track-Etched
Membranes. RSC Adv..

[49] Bush S. N., Volta T. T., Martin C. R. (2020). Chemical
Sensing and Chemoresponsive
Pumping with Conical-Pore Polymeric Membranes. Nanomaterials.

[50] Keesom W. H., Zelenka R. L., Radke C. J. (1988). A Zeta-Potential Model for Ionic
Surfactant Adsorption on an Ionogenic Hydrophobic Surface. J. Colloid Interface Sci..

[51] Ito T. (2023). Single-Molecule
Fluorescence Investigations of Solute Transport Dynamics in Nanostructured
Membrane Separation Materials. J. Phys. Chem.
B.

[52] De
Santo I., Causa F., Netti P. A. (2010). Subdiffusive Molecular
Motion in Nanochannels Observed by Fluorescence Correlation Spectroscopy. Anal. Chem..

[53] Xu H., Nagasaka S., Kameta N., Masuda M., Ito T., Higgins D. A. (2016). Imaging Fluorescence
Correlation Spectroscopy Studies
of Dye Diffusion in Self-Assembled Organic Nanotubes. Phys. Chem. Chem. Phys..

[54] Chu C. H., Sarangadharan I., Regmi A., Chen Y. W., Hsu C. P., Chang W. H., Lee G. Y., Chyi J. I., Chen C. C., Shiesh S. C., Lee G. -B., Wang Y. L. (2017). Beyond the Debye
Length in High Ionic Strength Solution: Direct Protein Detection with
Field-Effect Transistors (FETs) in Human Serum. Sci. Rep..

[55] Khamaisi B., Vaknin O., Shaya O., Ashkenasy N. (2010). Electrical
Performance of Silicon-on-Insulator Field-Effect Transistors with
Multiple Top-Gate Organic Layers in Electrolyte Solution. ACS Nano.

[56] Duan C., Majumdar A. (2010). Anomalous Ion Transport in 2-Nm Hydrophilic Nanochannels. Nat. Nanotechnol..

[57] Apel P. Y., Velizarov S., Volkov A. V., Eliseeva T. V., Nikonenko V. V., Parshina A. V., Pismenskaya N. D., Popov K. I., Yaroslavtsev A. B. (2022). Fouling
and Membrane Degradation in Electromembrane and Baromembrane Processes. Membr. Membr. Technol..

[58] Zhao C., Nie S., Tang M., Sun S. (2011). Polymeric PH-Sensitive Membranes
- A Review. Prog. Polym. Sci. (Oxford).

[59] Green Y. (2022). Effects of
Surface-Charge Regulation, Convection, and Slip Lengths on the Electrical
Conductance of Charged Nanopores. Phys. Rev.
Fluids.

[60] Cussler, E. L. Diffusion: Mass Transfer in Fluid Systems, 3rd ed.; Cambridge University Press: Cambridge, 2009 10.1017/CBO9780511805134.

[61] Mulder, M. Basic Principles of Membrane Technology, 2nd ed.; Springer Netherlands: Dordrecht, 1996. 10.1007/978-94-009-1766-8.

[62] Baker, R. W. Membrane Technology and Applications, 4th ed.; Wiley, 2024. 10.1002/0470020393.

[63] Barrett A., Imbrogno J., Belfort G., Petersen P. B. (2016). Phosphate Ions Affect
the Water Structure at Functionalized Membrane Surfaces. Langmuir.

[64] Schneider S., Brodrecht M., Breitzke H., Wissel T., Buntkowsky G., Varol H. S., Brilmayer R., Andrieu-Brunsen A., Vogel M. (2022). Local and Diffusive Dynamics of LiCl Aqueous Solutions in Pristine
and Modified Silica Nanopores. J. Chem. Phys..

[65] Steinrücken E., Diehl L., Wissel T., Buntkowsky G., Varol H. S., Andrieu-Brunsen A., Vogel M. (2025). Effects of Amino-Acid
Functionalization and PH Value on Temperature-Dependent Water Dynamics
in Silica Confinement. J. Chem. Phys..

[66] Gonçalves F. A., Kestin J. (1979). The Viscosity of CaCl2 Solutions in the Range 20–50
°C. Ber. Bunsen-Ges. Phys. Chem..

[67] Miles B. N., Ivanov A. P., Wilson K. A., Dogan F., Japrung D., Edel J. B. (2013). Single Molecule Sensing with Solid-State Nanopores:
Novel Materials, Methods, and Applications. Chem. Soc. Rev..

[68] Dong C., Ren J. (2014). Coupling of Fluorescence Correlation Spectroscopy with Capillary
and Microchannel Analytical Systems and Its Applications. Electrophoresis.

