# Supplementary material for: Modulating Electrostatic Interactions to Control the Analyte Transport in Nanochannels
Source: ACS Appl Mater Interfaces. 2025 Oct 6;17(41):57667–77. doi: 10.1021/acsami.5c14563 (PMC12532097; doi:10.1021/acsami.5c14563)
Supplement: Supplementary file 1 [file am5c14563_si_001.pdf]

Supporting Information

For

Modulating Electrostatic Interactions to Control the Analyte Transport in Nanochannels

H.Samet Varol,[1*] Matteo Cingolani,[1] Francesco Casnati,[1] Damiano Genovese[1*]

[1]*Dipartimento di Chimica"Giacomo Ciamician", Università di Bologna, via Selmi 2, Bologna, 40126, Italy*

Corresponding Authors(*): samet.varol@unibo.it; damiano.genovese2@unibo.it

Supplementary Methods:

Steady-state UV-VIS and Fluorescence Spectroscopy measurements of $Ru(bpy)_3^{2+}$ /PBS solutions. For both measurements, 5µM $Ru(bpy)_3^{2+}$ /PBS solutions at a fixed pH (pH 2.5 or pH 7) were placed inside PMMA disposable cuvettes (BRAND GMBH + CO KG) with 4 windows. The absorbance (UV/VIS) spectra were recorded using a PerkinElmer Lambda 650 UV-VIS spectrometer and together with the $Ru(bpy)_3^{2+}$ /PBS solution, a reference PBS solution (w/o dye) was also placed in the spectrophotometer. The spectra were collected between 300nm – 800nm. The steady-state fluorescence emission spectra of the 5µM $Ru(bpy)_3^{2+}$ /PBS solutions inside the PMMA cuvettes were recorded using a Fluoromax-4 Spectrofluorometer. The excitation wavelength (λ_{ext}) was 450nm, and both excitation and emission slit openings were 5nm. The emission spectra were collected between 500nm – 800nm. Both spectra were analyzed using a data processing software Igor Pro.

Calibration curves by using stead-state Fluorescence Spectroscopy measurements of different concentration $Ru(bpy)_3^{2+}$ /PBS solutions. 5mL total volume PBS (1X) solution was placed in a glass beaker with a magnetic stirrer and connected to our flow-through cuvette with the peristaltic pump (working at 120 rpm). The amount of $Ru(bpy)_3^{2+}$ gradually increased in this 5mL solution while collecting the emission spectrum from the solution inside our flow-through cuvette. For each analyte concentration from different pH solutions, we collected a minimum of 3 independent spectra. Then a linear fit (blue line in Figure S3b) was done for all the data (pH 2.5 and pH 7) between 0nmol and 60nmol.

Ethanol conditioning prior to the nanochannel transport of $Ru(bpy)_3^{2+}$. To perform diffusion experiments with ethanol (EtOH) conditioning, after a large nanochannel (LNC) membrane was placed in the diffusion setup, the whole setup was oriented vertically (the feed container was facing up). Then 250µL of EtOH was added on the feed side and directly on the membrane surface with the help of a plastic pipette and left in contact with the membrane for 5 min and then removed. Subsequently, 4mL of MilliQ water was added to both containers of the setup and left for a further 5min of conditioning to remove the excess of EtOH. Afterward, the usual ionic diffusion experiment was initiated by adding PBS (1X) solutions at pH 2.5 to the feed and permeate containers (see Methods for details).

Automated measurement of cationic mass transport (diffusion) using MiliQ as solvent and/or using NaCl as competitor monovalent ions. Differently from the automated nanochannel measurements that use 5µM $Ru(bpy)_3^{2+}$ /PBS (1X) solution as feed and pristine PBS (1X) solution (0mM $Ru(bpy)_3^{2+}$) as permeate, we used pure MiliQ (no PBS). For the measurements to understand the role of monovalent ion (NaCl) presence on $Ru(bpy)_3^{2+}$ transport, the feed solution (prior to any $Ru(bpy)_3^{2+}$ addition) contained different NaCl concentrations: 0 mM (pure Milli-Q water, no NaCl), 137 mM (standard 1X PBS, without additional NaCl), or 500 mM (1X PBS with extra NaCl added). After adjusting the pH, the solution was placed in the feed chamber, while the permeate chamber was filled with pristine 1X PBS at the same pH. Following the procedure described in the previous section, $Ru(bpy)_3^{2+}$ was added to the feed solution after 30 minutes of conditioning. In these diffusion measurements, 25 cycles (first 5 cycles for conditioning) of fluorescence emission acquisition were performed instead of 35 cycles. However, the rest of the experimental protocol was identical to those presented in Methods for experiments with PBS (1X) solutions.

2D and 3D Confocal Microscopy imaging. After 1h incubating both Small and Large nanochannel membranes (cut in 10mm diameter circular shapes) in 5µM $Ru(bpy)_3^{2+}$ solutions at high or low pH, the membranes were subsequently washed fast (5sec) of long (1h) washing steps inside similar pH pristine PBS (1X) solutions. Then, the samples were left inside the fume hood overnight for drying. Membranes were then imaged by a confocal microscope (Nikon Ti2 Inverted microscope with A1R HD laser-scanning) at similar lab conditions and using identical imaging parameters as described in the Methods section. The images were analyzed by ImageJ. For the 3D z-stack confocal images, two lasers were used, one at 489nm with Laser Power at 5% and detection in the channel 595 ± 50 nm with Photomultiplier Voltage (PMT HV) at 185V and the other at 402nm with Laser Power at 50% and detection in the channel 525 ± 50 nm with Photomultiplier Voltage (PMT HV) at 255V. The size of the image recorded is 1024x1024, and the intensity was averaged 8x. Ultimately, the full nanochannel length of the Large Nanochannel membrane was imaged with 3D stack images using a step of 0.5um over a 10um range.

Relating the Fitted Time Constant (τ) to Permeability (*P*): A Short Derivation for Equal-Volume Side-by-Side Cells.

Aim is to show that in an equal-volume, well-stirred side-by-side cell the fitted exponential time constant τ is inversely proportional to the membrane permeability *P*, and thus

$P_{pH\ 2.5}/P_{pH\ 7} = \tau_{pH\ 7}/\tau_{pH\ 2.5}$.

Two well-stirred chambers (feed → donor and permeate → receiver), each volume *V*; membrane area A; permeability *P* (units $m \cdot s^{-1}$); concentrations $C_d(t), C_r(t)$.

Classical flux law: $J = P(C_d - C_r)$ (per-area flux). Total molar rate across the membrane:

$$\dot{n} = A \cdot J.$$ [1–3]

Mass balances (equal volumes):

$$V\frac{dC_r}{dt} = +PA(C_d - C_r), \qquad V\frac{dC_d}{dt} = -PA(C_D - C_r).$$

Define the concentration difference $\Delta(t) \equiv C_d - C_r$. Subtracting the two differential equations:

$$\frac{d\Delta}{dt} = -\frac{2PA}{V}\Delta \implies \Delta(t) = \Delta(0)e^{-\frac{t}{\tau}}, \qquad \tau = \frac{V}{2PA}$$

Direct ratio for pH 2.5 and pH 7 (identical geometry): Since the V and A are the same in conditions pH 2.5 and pH 7, then

$$\frac{P_{pH\ 2.5}}{P_{pH\ 7}} = \frac{\tau_{pH\ 7}}{\tau_{pH\ 2.5}}$$

Quantification of nanochannel surface site occupancy by Ca^{2+} ions. To estimate the fraction of deprotonated carboxylate (COO^-) groups occupied by Ca^{2+} ions at deprotonated nanochannel walls (n_{Ca}) (Figure S6b), we used the total amount of $Ru(bpy)_3^{2+}$ transported across the membrane (T_{Ru}, see Figure 4b) at different $CaCl_2$ concentrations data ($T_{Ru,[Ca]}$, roughly proportional to the diffusion rate in the linear portion of the diffusion plot; see Methods and Supplementary; Figure 4b). Specifically, occupied binding sites in deprotonated nanochannels of LNC membrane via competing divalent Ca^{2+} ions at high pH (pH 7) at different concentrations of $CaCl_2$ ($[Ca^{2+}]$),was calculated using the following formula,

$$n_{Ca} = 1 - [\frac{T_{Ru,[Ca]} - T_{D,0}}{T_{Ru,max} - T_{D,0}}]$$

assuming that $n_{total} = n_{Ru} + n_{Ca}$ and $T_{Ru} \propto n_{total}^{-1}$, where T_{Ru} denotes the diffusion rates of $Ru(bpy)_3^{2+}$ in presence of varying $[Ca^{2+}]$, while n_{Ru} and n_{Ca} represent the number of binding sites occupied by $Ru(bpy)_3^{2+}$ and by Ca^{2+}, respectively. These values represent the estimated population of surface COO^- groups neutralized (occupied) by Ca^{2+} ions under each experimental condition.

Langmuir Isotherm fittings. Presented data in Figure S6 were analyzed using the Langmuir isotherm model, fitting the experimental (a) normalized average red color intensity or (b) fraction of deprotonated surface groups (COO^-) occupied binding sites by Ca^{2+} (both are presented below as I) data as a function of ligand ($Ru(bpy)_3^{2+}$ or $CaCl_2$) concentration $[L]$ to the equation:

$$I = I_{max} \cdot \frac{[L]}{K_D + [L]}$$

where I_{max} is the maximum I (norm. avg red color intensity or fraction of occupied binding sites by Ca^{2+}) at saturation, and K_D is the equilibrium dissociation constant.

"**Imaging and analysis of $Ru(bpy)_3^{2+}$ stained membranes dried at different vacuum drying durations.** Minimum three polycarbonate (LNC, see Methods) membranes were cut into square shape (5mm*5mm) and were incubated for 1 h in a 5 µM solution of $Ru(bpy)_3^{2+}$ prepared in 1X PBS at pH 7. After incubation, they were briefly washed for 5 seconds in analyte-free PBS (1X, pH 7, fast washing) to remove any unbound dye. The membranes were then dried under vacuum (≈10^{-2} mbar) for different durations: 1 h, 3 h, 24 h, and 1 week. After each drying interval, membrane fluorescence emission was recorded using the same photographic setup and imaging protocol described in the Methods section of the main manuscript. Briefly, membranes were illuminated under 365 nm UV light, and images were acquired using a ThorCam DCC1645C CMOS camera equipped with an HR f/1.4 22 mm lens and a 530 nm long-pass filter. Image acquisition settings (7 FPS, 140 ms exposure) and camera positioning were kept constant. Red-channel intensity was quantified using ImageJ, averaging the signal within a circular ROI for each membrane and normalizing to the red intensity of non-stained reference membranes.

Fluorescence Lifetime Measurements via Time Correlated Single Photon Counting (TCSPC). Dried membranes after 1 h, 3 h, 24 h, and 1-week drying under vacuum, $Ru(bpy)_3^{2+}$ powder (see Methods), pristine LNC membrane (not exposed to any $Ru(bpy)_3^{2+}$ solution) were sandwiched between two microscope glass cover slips (24x40x0.13-0.17mm, Prestige) and then placed diagonally across the excitation–emission beam path for optimal overlap and signal collection during TCSPC measurements. Fluorescence lifetime data were recorded using an Edinburgh FLS920 spectrometer TCSPC setup equipped with a pulsed diode laser operating at λ_{exc} = 450 nm. Emission was collected through a monochromator centered at λ_{em} = 608 nm and detected by a detector. The lifetime decay profiles were analyzed using a biexponential decay model:

$$I(t) = B_1 e^{-t/\tau_1} + B_2 e^{-t/\tau_2}$$

where $\boldsymbol{\tau_1}$ and $\boldsymbol{\tau_2}$ are the characteristic lifetimes, and $\mathbf{B_1}$ and $\mathbf{B_2}$ are their respective amplitudes. Fitting was performed using the built-in software of the device. The precision of the fit was

assessed using χ^2 values and residuals. Lifetime values were reported as intensity-weighted average fluorescence lifetime (τ_{int}) by applying the following formula,

$$\tau_{int,avg} = \frac{\sum_{i=1}^{3} B_i \tau_i^2}{\sum_{i=1}^{3} B_i \tau_i}$$

The $\tau_{int,avg}$ values and standard deviations (sd) at each drying duration (Table S1) were determined from three independent dipping experiments at each condition."

Supplementary Figures:

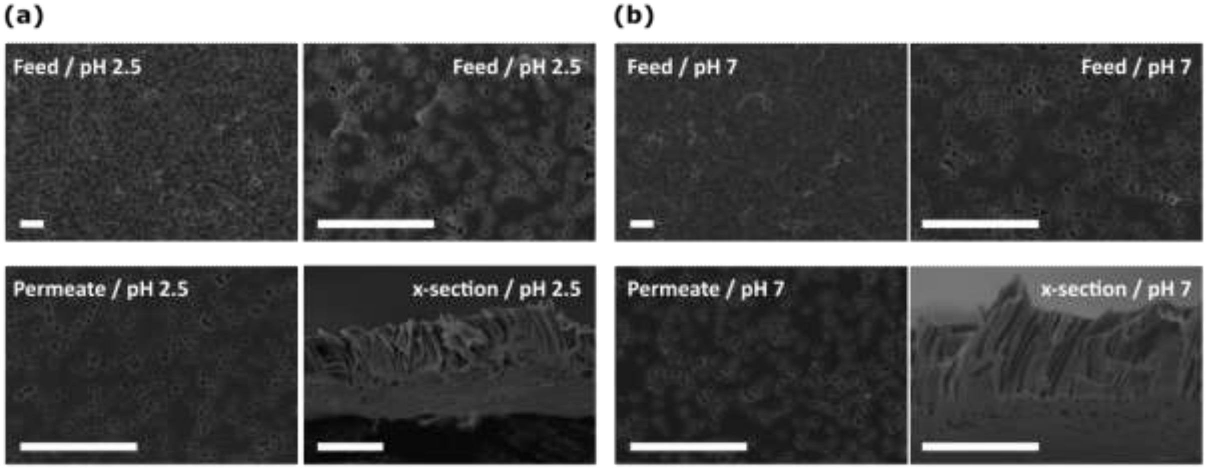

Figure S1. SEM micrographs of the Large Nanochannel Membranes after their 3.5h $Ru(bpy)_3^{2+}$ diffusion tests performed at (a) pH 2.5 and (b) pH 7. Scale bars are 10µm.

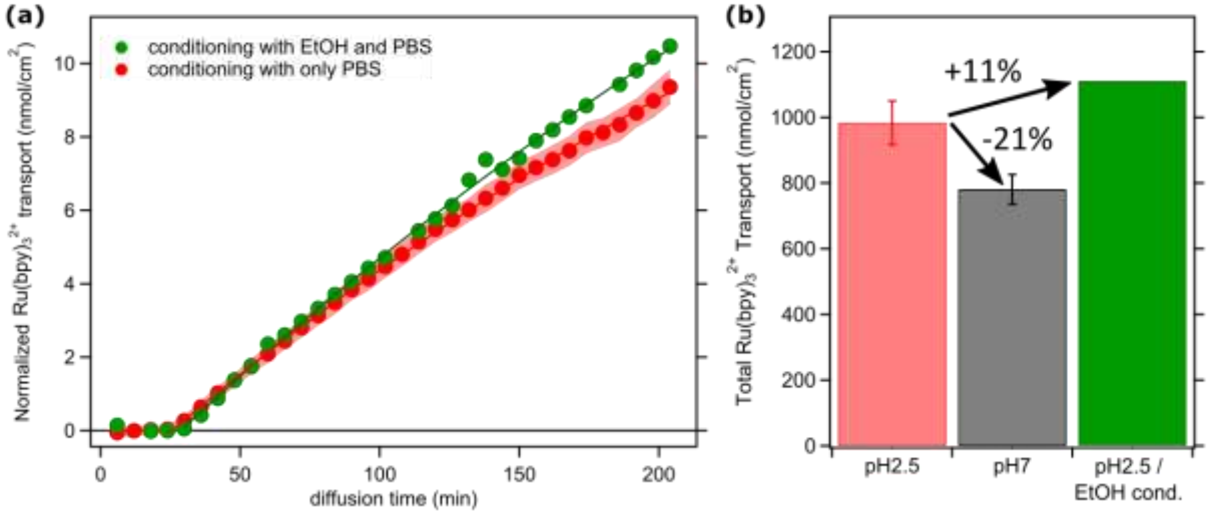

Figure S2. (a) Normalized analyte ($Ru(bpy)_3^{2+}$ (nmol/cm^2)) diffusion through Large Nanochannel Membranes at pH2.5 after conditioning the membrane nanopores with only PBS (red filled markers), or first by ethanol and then PBS (green). Solid green and red lines are the exponential fit of the presented diffusion results (filled-circle markers). (b) Total amount of $Ru(bpy)_3^{2+}$ dye diffused to the permeate cell within 3h of diffusion at different pH and conditioning conditions. Error bars are sd.

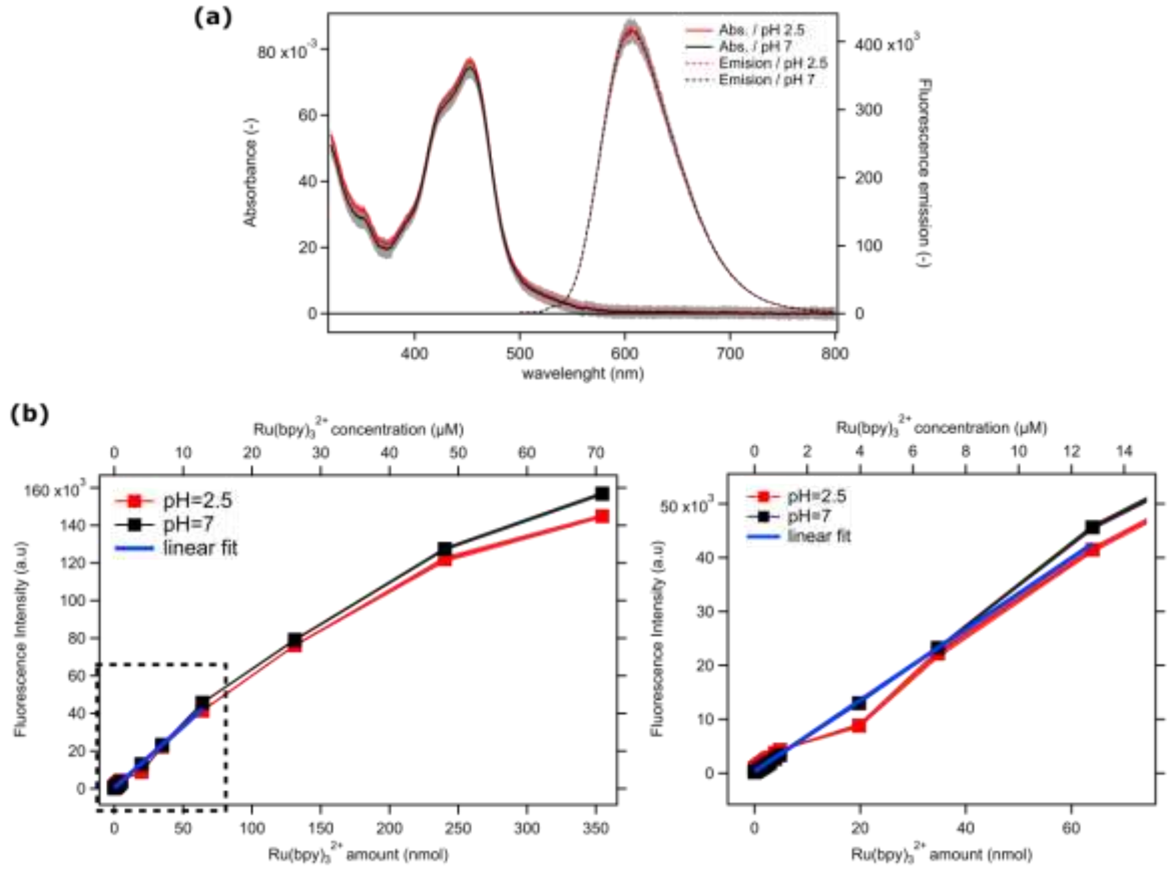

Figure S3. (a) UV-VIS and Fluorescence emission spectra of 5µM of $Ru(bpy)_3^{2+}$ /PBS solutions at different pH. (b) Calibration curves presenting the relation between the fluorescence intensity of the emission peak at 605nm and the $Ru(bpy)_3^{2+}$ amount (nmol), and concentration (µM) at pH2.5 and pH7, and the linear calibration fit (blue solid line) was fitted for the data collected between 0nmol and 60nmol. The graph in the right is the magnified portion of the calibration graph on the left indicated by black dashed line box. Shaded area are sd derived from the peak intensity collected for (a) from 3 and for (b) from 6 independent spectra (during kinetic measurements; see Methods for more details) collected from the same solution.

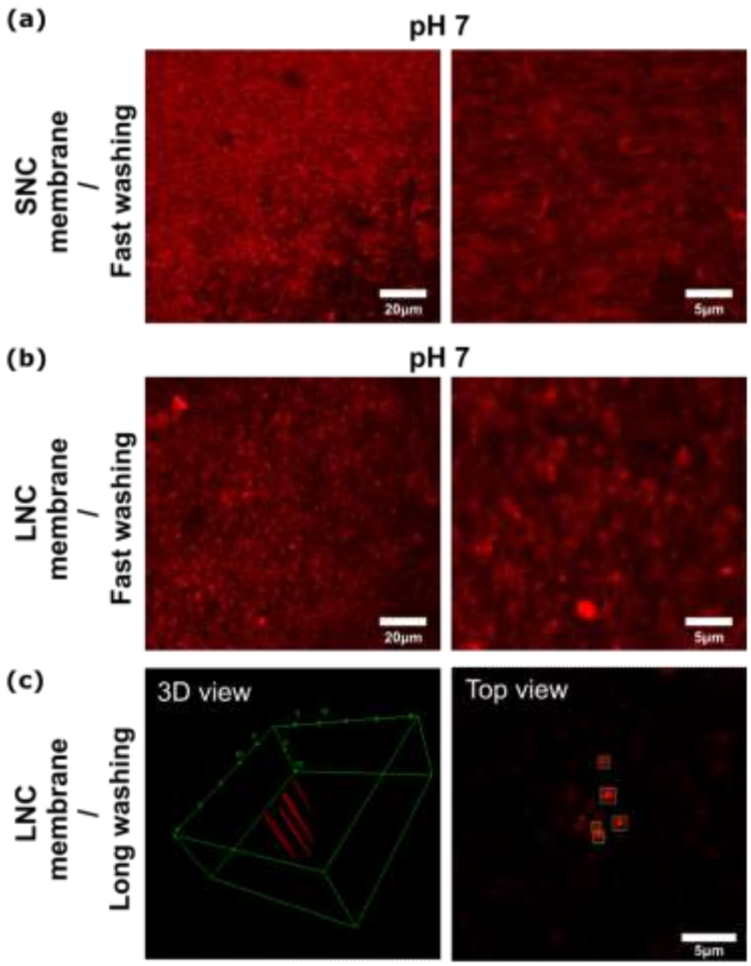

Acquisition Method	$I\,(red)_{pH2.5}/I\,(red)_{pH7}$ LNC membrane	SNC membrane
**Photographic images** by average *red* channel *intensity*	0.51±0.20	0.43±0.28
**Confocal microscopy** images by *raw integrated density*	0.82±0.16	0.25±0.11

Figure S4. (a-b) Low (left) and high (center and right) magnification 2D Confocal images of the dry (a) small nanochannel (*SNC*) and (b) large nanochannel (*LNC*) membranes that were dipped inside the 5 µM $Ru(bpy)_3^{2+}$ solution at high and low pH for 1h and then washed inside only PBS solution (no dye, similar pH as dye solution) for 5s (fast washing). (c) (left) Confocal 3D z-stack image and (right) a single XY-orthoslice image (from the z-stack) from the center thickness of the large nanochannel membrane sample that was washed for a long period (1h) after dye incubation (5 µM $Ru(bpy)_3^{2+}$). (left) Numbers on the green border box present the scales in µm. (d) Ratio ($I\,(red)_{pH2.5}/\,I(red)_{pH7}$) of the collected red-light intensity from the remaining $Ru(bpy)_3^{2+}$ analyte at the membrane surface after they were dipped inside different pH analyte solutions and analyzed by using their photographic images (Figure 3a) or 2D confocal images (a-b). Errors are sd from a minimum two figures from each experimental conditions.

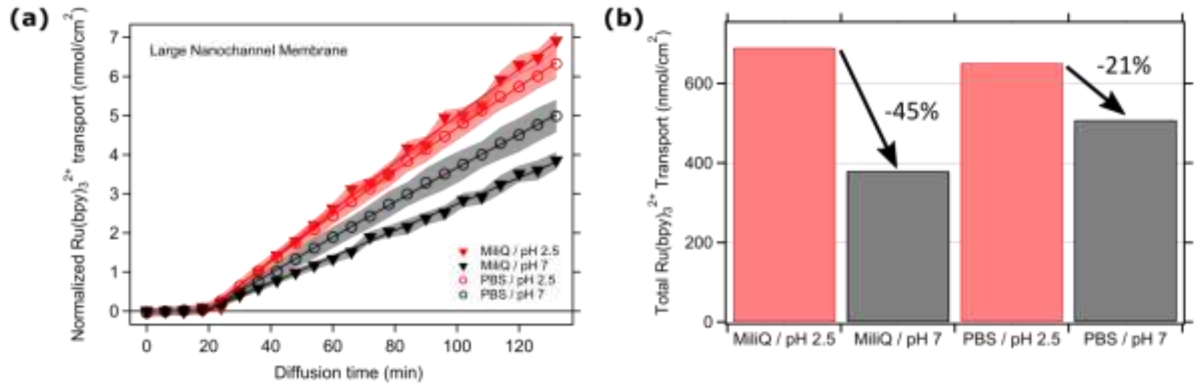

Figure S5. (a) Normalized analyte ($Ru(bpy)_3^{2+}$ ($nmol/cm^2$)) diffusion at Large Nanochannel Membranes at high and low pH while only MiliQ water (filled-triangles) or MiliQ water with PBS (1X) (empty circles) were used as solvents. Solid lines are exponential fits, and shaded regions are the sd derived from a minimum of three measurements for each condition. (b) Total amount of $Ru(bpy)_3^{2+}$ dye transported through the large nanochannel membrane after the 2h 12min of diffusion at different pH and solvents.

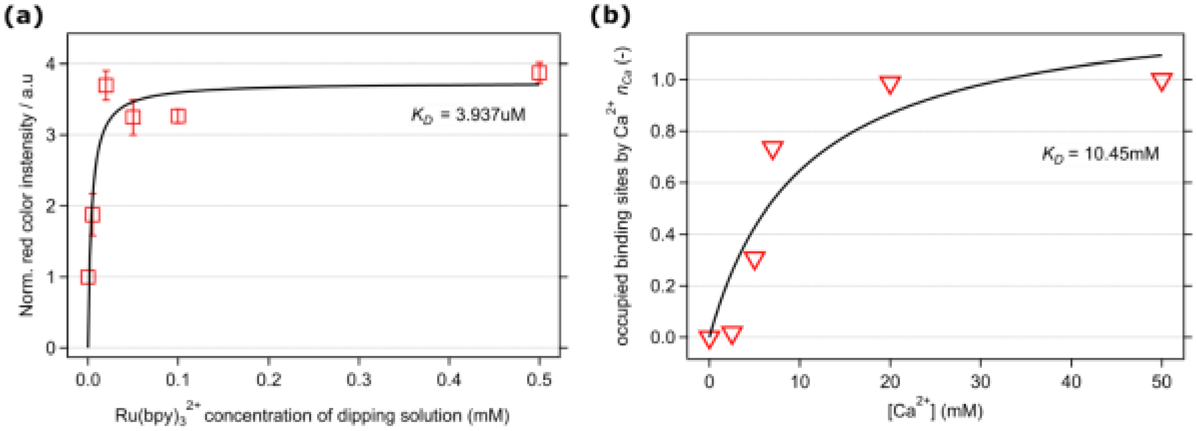

Figure S6. (a) Normalized average red color intensity detected from the LNC membranes after they were dipped in 0mM, 0.05mM, 0.02mM, 0.05mM, 0.1mM, and 0.5mM of $Ru(bpy)_3^{2+}$ solutions (1X PBS, pH7) and subsequently washed in 0mM PBS (1X, pH7) solution for 5sec (fast washing). After washing, the membranes were left under vacuum for a minimum of 1 hour for drying. Error bars are sd generated from the photo of at least three independent membranes dipped inside each $Ru(bpy)_3^{2+}$ solution. (b) Occupied binding sites in the deprotonated LCN nanochannels by Ca^{2+} ions at pH 7 and different concentrations of $CaCl_2$ (n_{Ca}), as obtained by using total $Ru(bpy)_3^{2+}$ transport data (T_{Ru}) by following formula $n_{Ca} = 1 - [(T_{Ru,[Ca]} - T_{Ru,0}) / (T_{Ru,max} - T_{Ru,0})]$, assuming that $n_{total} = n_{Ru} + n_{Ca}$ and $k_D \propto n_{total}^{-1}$, where $T_{Ru,[Ca]}$ denotes the total $Ru(bpy)_3^{2+}$ transport in permeate chamber in presence of varying [Ca^{2+}], while n_{Ru}, n_{Ca} and n_{Total} represent the number of binding sites occupied by $Ru(bpy)_3^{2+}$, Ca^{2+}, and total number of binding sites at high pH, respectively,. Black lines represent the Langmuir adsorption model fit to the data presented in (a) and (b). Dissociation constants (K_D) are noted next to corresponding Langmuir fits.

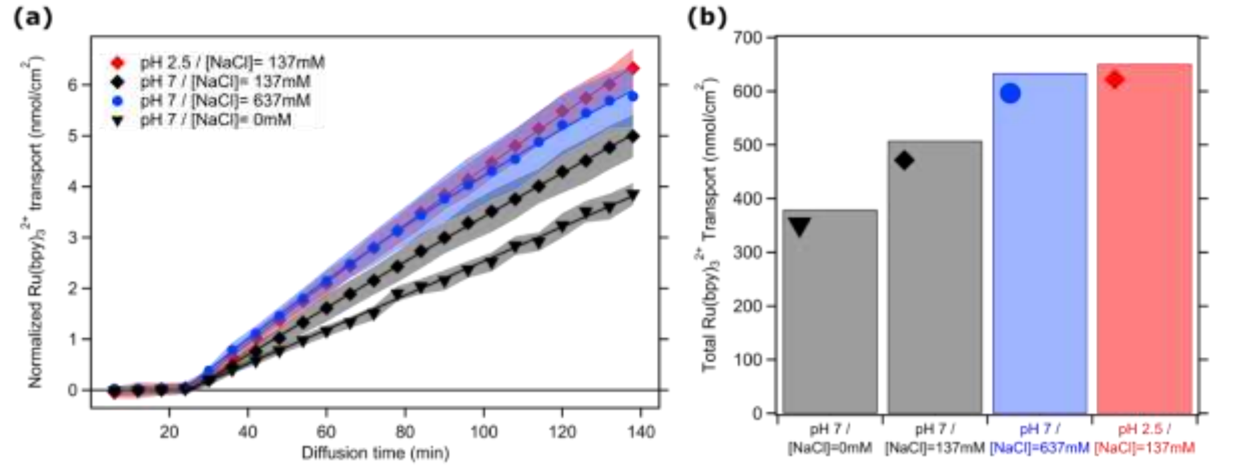

Figure S7. (a) Normalized transport of $Ru(bpy)_3^{2+}$ (nmol/cm²) inside the Large Nanochannel (LNC) membrane at pH 2.5 (red filled diamond markers) and pH 7 (black filled diamond markers) in 1x PBS ([NaCl] = 137 mM, black), at pH 7 with additional 500mM NaCl inside the 1X PBS ([NaCl] = 637mM, blue filled circles) and in MiliQ water in the absence of any PBS (black upside down filled triangles). The results in red filled diamond and black upside-down filled black triangle markers are identical to those already presented in Figure S5. The straight lines inside the data markers are the exponential fits of the corresponding data in (a) (see Methods for details). The shed areas are sd and were obtained from at least three independent diffusion measurements at each specific condition. (b) Total amount of $Ru(bpy)_3^{2+}$ transported through the LNC membrane at different pH and amounts of NaCl presence. The data presented by the black bar (with upside-down filled black triangle) and the red bar are a reproduction of the same data presented in Figure S4b.

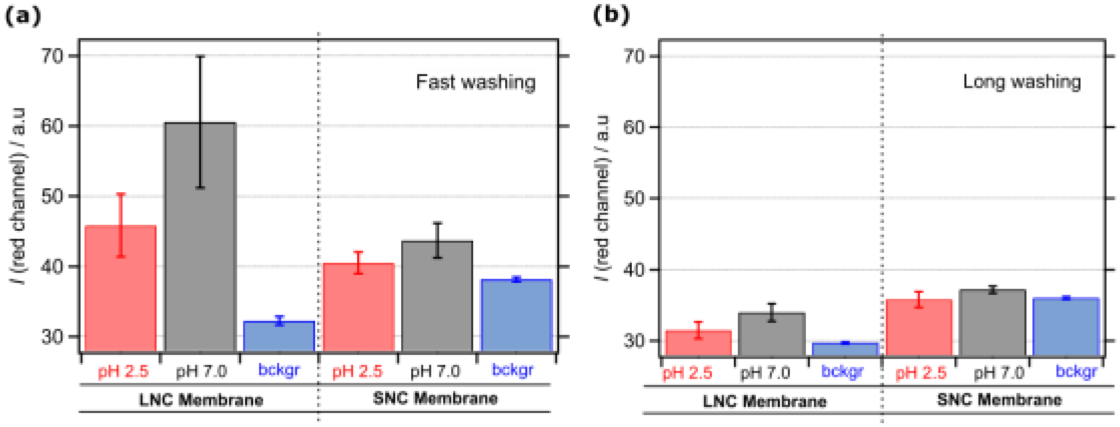

Figure S8. (a-b) Non (background) normalized red light intensities quantified from the photographic images (see insets in Figure3a-b) of our Large (left) and Small (right) Nanochannel Membranes after dipping them inside 5µM $Ru(bpy)_3^{2+}$ solutions with different pH. The dipped membranes were then washed inside only PBS (no dye) solutions for (a) 5 sec (fast washing) or (b) 1h (long washing). The photographic images of the dipped and washed samples were subsequently taken after illuminating the samples under 365nm light. Error bars in all the bar graphs are sd from three independent dipping experiments.

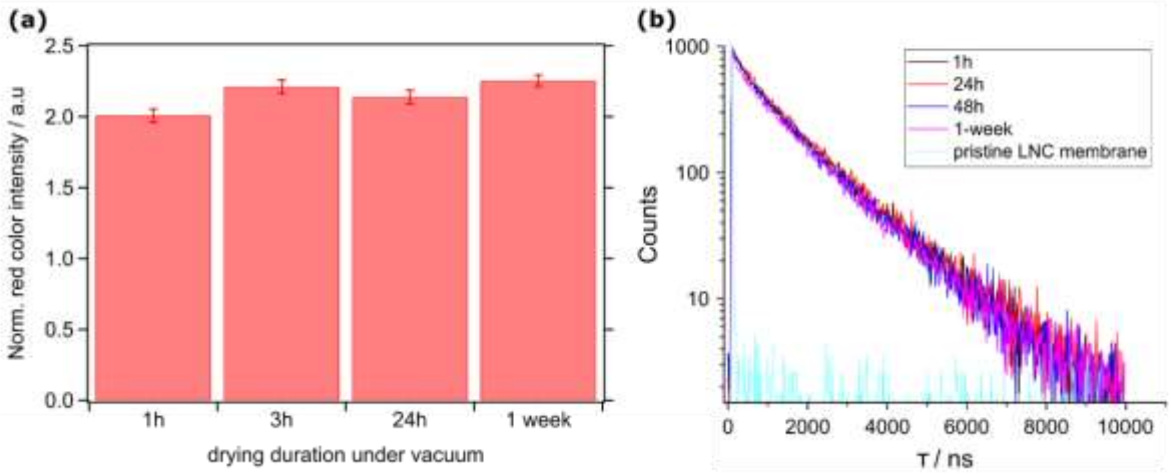

Figure S9. (a) Normalized red color intensity and (b) normalized fluorescent lifetime (τ) decays collected from the LCN membranes after they were dipped inside 5µM $Ru(bpy)_3^{2+}$ PBS solution (1X, pH 7), washed (in bare PBS solution (1X), pH7, for 5 sec) and left under high vacuum for drying at different time periods between 1h and 1-week. Error bars in (a) represent the standard deviation (SD) collected from three independent photoss taken from three different cut LNC membrane pieces (0.5 cm × 0.5 cm) at each of the different drying conditions. In (b), together with τ collected from the membranes after dipping and drying test, also τ from pristine LCN membrane (cyan) (not contacted any $Ru(bpy)_3^{2+}$ solution).

Table S1. Intensity average characteristic fluorescence lifetimes ($\tau_{int,avg}$) with standard deviations (± sd) calculated from minimum three samples from each dipping and vacuum drying condition. The LNC membranes were first dipped in a 5 µM concentration of $Ru(bpy)_3^{2+}$ (1X PBS, pH 7) and subsequently washed (fast, 5 sec). They were then left under vacuum for varying durations to dry. The values are derived from biexponential fitting of time-resolved measurements.

Drying duration under vacuum	$\tau_{int,avg}$ ± sd (ns)
1h	1350 ± 35
24h	1353 ± 33
48h	1313 ± 9.7
1-week	1333 ± 41

Supplementary References

(1) Cussler, E. L. *Diffusion: Mass Transfer in Fluid Systems*, 3rd ed.; Cambridge University Press: Cambridge, 2009. https://doi.org/https://doi.org/10.1017/CBO9780511805134.

(2) Mulder, M. *Basic Principles of Membrane Technology*, 2nd ed.; Springer Netherlands: Dordrecht, 1996. https://doi.org/10.1007/978-94-009-1766-8.

(3) Baker, R. W. . *Membrane Technology and Applications*, 4th ed.; Wiley, 2024. https://doi.org/10.1002/0470020393.
